# Selective Janus kinase 1 inhibition resolves inflammation and restores hair growth offering a viable treatment option for alopecia areata

**DOI:** 10.1002/ski2.209

**Published:** 2023-01-29

**Authors:** Johan Mattsson, Elisabeth Israelsson, Karin Björhall, Linda Fahlén Yrlid, Kristoffer Thörn, Anna Thorén, Emelie Andersén Toledo, Lisa Jinton, Lisa Öberg, Cecilia Wingren, Sofia Tapani, Sonya G. Jackson, Gabriel Skogberg, Anders J. Lundqvist, Ramon Hendrickx, Anders Cavallin, Torben Österlund, Neil P. Grimster, Magnus Nilsson, Annika Åstrand

**Affiliations:** ^1^ Bioscience, Research and Early Development Respiratory & Immunology (R&I) BioPharmaceuticals R&D AstraZeneca Gothenburg Sweden; ^2^ Translational Science and Experimental Medicine Research and Early Development Respiratory & Immunology (R&I) BioPharmaceuticals R&D AstraZeneca Gothenburg Sweden; ^3^ Animal Science and Technologies Clinical Pharmacology & Safety Sciences BioPharmaceuticals R&D AstraZeneca Gothenburg Sweden; ^4^ Early Biometrics & Statistical Innovation Data Science & AI BioPharmaceuticals R&D AstraZeneca Gothenburg Sweden; ^5^ Drug Metabolism & Pharmacokinetics Research and Early Development Respiratory & Immunology (R&I) BioPharmaceuticals R&D AstraZeneca Gothenburg Sweden; ^6^ The Discovery Sciences Unit BioPharmaceuticals R&D AstraZeneca Gothenburg Sweden; ^7^ Chemistry Oncology R&D AstraZeneca Waltham Massachusetts USA; ^8^ Medicinal Chemistry BioPharmaceuticals R&D AstraZeneca Gothenburg Sweden

## Abstract

**Background:**

Janus Kinase (JAK) inhibition has recently demonstrated therapeutic efficacy in both restoring hair growth and resolving inflammation in Alopecia Areata (AA). These effects are dose dependent and mainly efficacious at ranges close to a questionable risk profile.

**Objectives:**

We explored the possibility to separate the beneficial and adverse effects of JAK inhibition by selectively inhibiting JAK1 and thereby avoiding side effects associated with JAK2 blockade.

**Methods:**

The C3H/HeJ mouse model of AA was used to demonstrate therapeutic efficacy in vivo with different regimens of a selection of JAK inhibitors in regards to systemic versus local drug exposure. Human peripheral blood lymphocytes were stimulated in vitro to demonstrate translation to the human situation.

**Results:**

We demonstrate that selective inhibition of JAK1 produces fast resolution of inflammation and complete restoration of hair growth in the C3H/HeJ mouse model of AA. Furthermore, we show that topical treatment does not restore hair growth and that treatment needs to be extended well beyond that of restored hair growth in order to reach treatment‐free remission. For translatability to human disease, we show that cytokines involved in AA pathogenesis are similarly inhibited by selective JAK1 and pan‐JAK inhibition in stimulated human peripheral lymphocytes and specifically in CD8^+^ T cells.

**Conclusion:**

This study demonstrates that systemic exposure is required for efficacy in AA and we propose that a selective JAK1 inhibitor will offer a treatment option with a superior safety profile to pan‐JAK inhibitors for these patients.

## Graphical abstract

We have shown that selective JAK1 inhibition is as effective as pan‐JAK inhibitors in restoring normal immune signatures and hair growth, in mice and in human peripheral blood mononuclear cells as well as CD8+ T‐cells. Importantly, our data shows that systemic exposure is essential for efficacy and that prolonged treatment post restoration of hair growth is needed for immune privilege establishment and maintained remission.

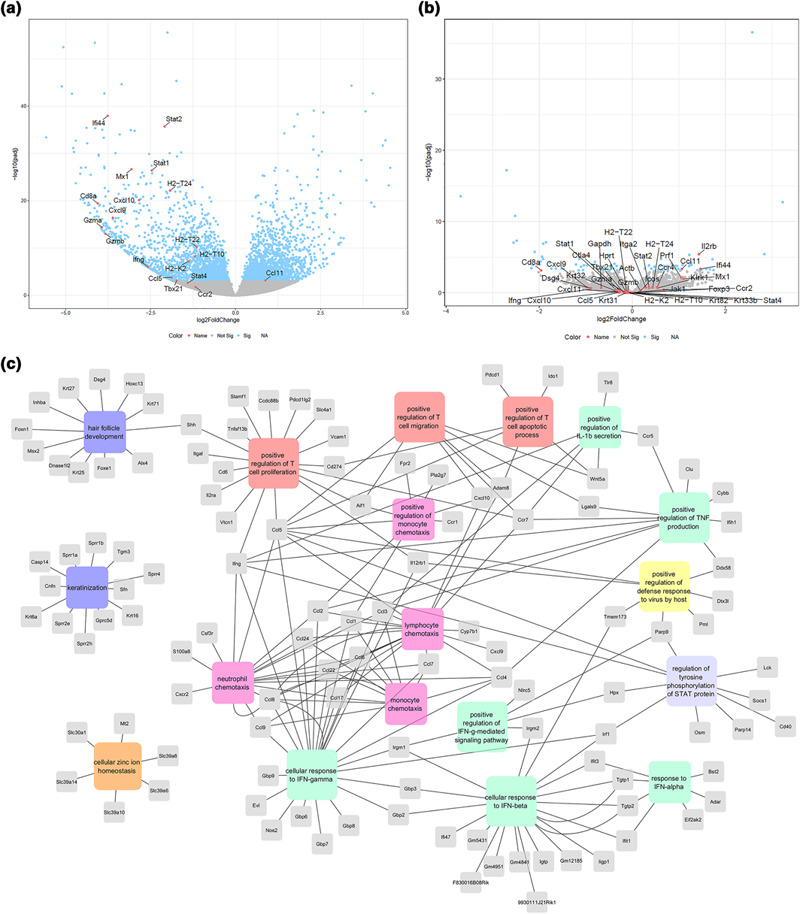

1

**What is already known?**

- Janus kinase (JAK) inhibitors have been shown efficacious in restoring hair growth in patients with Alopecia Areata.
- Relapses are common after cessation of treatment.
- Improved risk:benefit profile with JAK inhibitors is warranted.

**What does this study add?**

- Systemic JAK inhibition is needed for efficacy, topical/local inhibition is not sufficient unless it spills over systemically.
- Selective JAK1 inhibition is as efficacious as pan‐JAK inhibition.
- An extensive treatment period, beyond remission, is needed for maintained effect.

**What is the translational message?**

- Human T‐cells are as responsive to selective JAK1 inhibition as pan‐PAK inhibition with regards to cytokine release and gene expression profile known to be of importance in Alopecia Areata.
- In vivo effects by JAK inhibitors in the C3H/HeJ mouse model translate well into clinical efficacy.

## 2 INTRODUCTION

Alopecia Areata (AA) is a common autoimmune disease worldwide that affects men and women equally^1^{McElwee, 2013 #41}. The disease progression is unpredictable and can strike at any age but often manifests in children and adolescents (<30 years in >60% of the cases) and is frequently triggered by a stressful, traumatic or infectious period.^2^, ^3^ AA often starts as coin‐sized patches that spontaneously resolve but can progress to the more severe type, Alopecia Universalis (AU), where all body hair is attacked. Because of the nature of this non‐life‐threatening disease, finding a cure and optimizing treatment regimens is perceived as of low importance. However, AA patients have an increased risk of developing other autoimmune diseases such as atopic dermatitis, thyroid dysfunction, vitiligo, diabetes mellitus, rheumatoid arthritis, systemic lupus erythematosus, irritable bowel disease, psoriasis, psoriatic arthritis, besides psychological disorders like depression and anxiety.^4^, ^5^ This is not surprising given that there is a notable overlap in the underlying genetic predisposition in many of these diseases.^6^ Understanding and treating the underlying immune disorder would be beneficial in AA and could potentially also have a positive impact on other severe comorbidities.^7^, ^8^

Recently, several milestones in understanding the underlying mechanism in the manifestation and progression of AA have been achieved and this has accelerated the development of new and effective treatment options for AA patients. The involvement and importance of the JAK‐STAT (Janus Kinase‐Signal Transducer and Activator of Transcription) pathway was published by Xing et al. in 2014 showing efficacious treatment of AA mice as well as human patients with Tofacitinib (CP‐690,550, pan‐JAK inhibitor, Xeljanz) or Ruxolitinib (INCB018424, JAK1/JAK2 inhibitor, Jakafi).^9^ They presented a translational cytotoxic gene signature in mouse and human skin and demonstrated the importance of JAK signalling cytokines and NKG2D^+^ CD8^+^ T cells in AA. In fact, adoptive transfer of NKG2D^+^ CD8^+^ T cells from mice with AA to healthy recipients was sufficient to induce disease. The contribution of these cytotoxic cells in human disease has been further supported by the identification of SNP risk factors in the NKG2D ligands ULPB6, ULBP3 and MICA in an AA genome‐wide association study.^6^ Concomitant to disease induction is the loss of immune privilege (IP) of the hair follicles (HF). HF IP is normally characterized by low expression of major histocompatibility complex (MHC) class I molecules, NKG2D ligands as well as the upregulated expression of immunosuppressants.^10^, ^11^, ^12^ The loss of IP can be induced by inflammation, and IFN‐γ in particular, has been suggested to be an important trigger in the early stages of AA.^10^, ^13^ The self‐antigen that is recognized by the auto‐reactive T cells in AA remains elusive, although antigens such as trichohyalin and certain keratins, only expressed during the susceptible anagen (growth) phase, have been suggested as potential targets.^14^, ^15^, ^16^ Nonetheless, with the loss of IP, the induction of MHC I molecules and NKG2D ligands on HF cells make them vulnerable to attack by cytotoxic cell types such as CD8^+^ T cells and NK cells. However, only the base of the HF is affected, sparing the stem cells of the bulge region, resulting in non‐scarring and potentially reversible hair loss.^17^

Variable efficacy by different JAK inhibitors has since been demonstrated in several clinical studies and case reports^9^, ^18^, ^19^, ^20^, ^21^, ^22^, ^23^, ^24^, ^25^, ^26^, ^27^, ^28^, ^29^ with the observation that relatively high doses (equivalent to what is given in the treatment of rheumatoid arthritis) are required for adequate efficacy. Even though no serious side effects were reported by the use of ruxolitinib or tofacitinib in relatively small clinical studies, there is a previously described increased risk of respiratory infections due to the immunosuppressive effects following long term use. The safety profile for Baricitinib (10 and 100‐fold selectivity over TYK2 and JAK3, respectively^30^) in AA was recently presented to be similar to that found in RA and AD^31^ (Press release https://investor.lilly.com/node/44896/pdf). Moreover, the efficacy and safety of the JAK3/TEC inhibitor, ritlecitinib (PF‐06651600), has recently been released,^32^, ^33^ https://www.pfizer.com/news/press-release/press-release-detail/pfizer-announces-positive-top-line-results-phase-2b3-trial]. Thrombocytopaenia, anaemia and neutropenia, resulting from inhibition of the JAK2‐signalling thrombopoietin, erythropoietin and G‐CSF/GM‐CSF respectively, have been described,^34^, ^35^, ^36^ which promotes the notion that selectivity against JAK2 may be desirable to improve safety. Recently, an overview of the JAK/STAT pathways and JAK inhibition in AA was elegantly reviewed by Lensing and Jabbari which highlights the potential selectivity preferences for JAK inhibition in this disease.^37^ Evaluation of pulsed protocols, different induction and maintenance dosing regimens, as well as topical delivery have been proposed to minimize the risk profile of JAK inhibitors for the treatment of AA. Recently, a systematic meta‐analysis of prospective clinical trials of JAK inhibition in AA by Yan et al.^38^ demonstrated efficacious and generally well‐tolerated treatments with oral administration, whereas topical or sublingual administration lack efficacy. Encouragingly, the efficacy and safety of a number of JAK inhibitors in AA are currently under evaluation.^39^

Here, we hypothesized that selective JAK1 inhibition would be as efficacious as tofacitinib and ruxolitinib to reverse AA, as JAK1 inhibitors can block the signalling of CD8^+^ T cell survival factors such as IL‐2 and IL‐15 as well as the effector cytokine IFN‐γ, known to be central to the pathogenesis of AA. Moreover, we set to investigate whether local delivery without systemic exposure would be sufficient for efficacy, as that would also improve the safety risk profile of a potential drug.

## 3 MATERIALS AND METHODS

### 3.1 Study design

The objective was to investigate the efficacy of selective JAK1 inhibition in the C3H/HeJ mouse model of AA as both ruxolitinib and tofacitinib had demonstrated complete resolution of inflammation and restoration of hair growth in both mice and humans with AA. Moreover, to investigate whether local JAK1 inhibition (adequate exposure in the skin, but none in circulating plasma) would be an efficacious treatment regimen to further reduce the risk of the known systemic side effects driven by JAK1 inhibition, we used small molecule JAK1 inhibitors with either good bioavailability after oral administration for systemic exposure or with excellent tissue retention, dosed topically by means of a transdermal cream formulation, for local exposure only.^40^, ^41^, ^42^ To translate the efficacy and potential for selective JAK1 inhibition in clinical practice for the treatment of AA, we compared concentration‐dependent action of ruxolitinib^43^ and tofacitinib^44^ to a selective JAK1 inhibitor in human peripheral blood mononuclear cells (PBMCs) and cytotoxic CD8^+^ cells, with regards to cytokine release and mRNA expression.

### 3.2 Chemical tools

The physiochemical properties of tofacitinib,^44^ ruxolitinib^43^ and the three selective JAK1 tool compounds^40^, ^41^, ^42^ were thoroughly investigated by means of potency, selectivity and pharmacokinetic profiles.

#### 3.2.1 Kinase inhibition

JAK inhibition and selectivity of the JAK inhibitors was determined at physiological conditions in a Calliper assay as previously described.^41^

#### 3.2.2 Cell assays

For functional cell assays we employed a U937 stable cell line expressing the firefly luciferase gene controlled by three STAT6 response elements. The U937 cell is known to respond strongly to both IL‐4 and IL‐13 inducing STAT6 phosphorylation and activation, and dose‐response curves were obtained for these interleukins. Both EC_50_ and EC_80_ values were determined and the interleukin concentration inducing 80% of Emax (EC_80_) activity was used for inhibition assays. JAK inhibitors were tested in dose‐response assays and the inhibitory dose‐response curves were used for determination of IC_50_ values for the JAK inhibition.

#### 3.2.3 Pharmacokinetic profile

Male C57Bl6 mice were dosed either intravenously (0.5–1 mg/kg) or by gavage (1–300 mg/kg) and blood was sampled from the metatarsal vein repeatedly from 2 min to 24 h after administration to acquire pharmacokinetic properties such as oral bioavailability, plasma clearance etc for each compound. Satellite animals treated via the same route of administration as the study animals were run upfront to set adequate doses and establish exposure profiles for the study animals. Plasma protein binding was obtained by equilibrium dialysis to link potency and efficacy to free fractions of compound in the different compartments, blood and skin (using the Gillette equation for skin exposures). Detailed description in Supplementary materials.

### 3.3 In vivo experiments

All preclinical procedures were approved by the local Ethical committee in Gothenburg.

#### 3.3.1 Mice

Female C3H/HeJ graft‐induced mice were used as a preclinical model of Alopecia Areata (AA) according to previous publications.^45^, ^46^, ^47^ Healthy C3H/HeJ and mice with fully developed AA (16–24 weeks after induction of disease) were monitored by means of body weight and hair index score on a weekly basis for 12 weeks, with free access to food and water (Healthy and AA groups, respectively).

#### 3.3.2 Hair scoring

Hair growth was evaluated using a scoring index (Hair Index Score, HIS). A score from 0 to 3 (0 = no fur, 1 = stubble, short broken hairs, 2 = sparse, intermediate length hairs, 3 = normal length and density hairs) was taken times the surface area of the mouse with each respective score (dorsal area = 50%, ventral area = 50%). A HIS of 0 indicated no fur and a score of 300 demonstrated complete, normal densed hair.

#### 3.3.3 Treatments and groups

12 mice were given once daily servings of Cmpd A for free eating at approximately 100 mg/kg (compound dissolved in 10 ml tap water and mixed with 5 g grounded R70 standard rodent chow from Lantmännen, Sweden) for 4 weeks. Typically, these servings were finished within 2 h. Six mice were terminated at the end of the treatment period, blood and skin taken for determination of drug concentration and skin sampled for histopathological evaluation (see below, these mice are representing the systemic JAK1 inhibition group in histopathological evaluations). Skin biopsies for mRNA expression and blood was sampled from the other 6 mice where after these mice were followed off treatment for an additional 8 weeks when terminal samples were taken (8w off Tx group).

Another set of 6 mice were treated topically on the right side of their back with Cmpd B (0.1% cream formulation, Eucerin Aquaporin rich) for 12 weeks (local JAK1 inhibition group). Initially, twice weekly for 2 weeks followed by once weekly due to high tissue retention. Plasma exposure was evaluated during the course of the study as well as at termination when also skin was sampled for exposure, histopathological evaluation and mRNA expression.

In order to confirm the systemic versus local in vivo experiments with Cmpd A and B, we initiated a study with Cmpd C that exhibit the right properties required for both oral delivery and topical application. The aim of the study was to achieve similar skin exposures by both routes of administration but only systemic exposure after oral administration. Six mice were treated once daily with oral servings as described above (12.5 mg/kg/day, systemic JAK1 inhibition group) and 6 mice treated topically once daily (0.01% cream formulation, local JAK1 inhibition group) for 2 weeks to reach steady state efficacy and achieve a clear readout of hair growth. Blood, skin, lymph nodes and spleens were collected at termination for evaluation of exposure, mRNA expression, histopathology (RNAScope) and cell differentials (flow cytometry). Untreated healthy and alopecic mice served as controls (*n* = 6). In a separate set of experiments, AA mice were treated topically with 3% cream formulation of tofacitinib (*n* = 5).

#### 3.3.4 Plasma and skin exposures of compound A, B, C & tofacitinib

Compound concentrations in plasma samples were determined by liquid chromatography‐tandem mass spectrometry (LC‐MS/MS). Briefly, a gradient elution on a C18 column was used with acetonitrile/formic acid as the mobile phase system. The mass spectrometer was operating in a positive/negative switching mode. Plasma samples were protein precipitated by acetonitrile and diluted after centrifugation. Skin tissue samples were pulverized in a CryoPrep (Covaris) and homogenized with Ringer solution. The homogenate was protein precipitated prior to analysis by LC/MSMS using the same protocol as for plasma samples. Detailed description in Supplementary materials.

#### 3.3.5 Analysis of circulating cytokines in mouse plasma

A murine panel of 11 cytokines were analyzed in plasma samples from healthy, AA and mice treated with Cmpd C. A Luminex assay kit from Merck Millipore (Cat# MTH17MAG‐47K) was used and the analysis carried out according to the supplier protocols.

#### 3.3.6 Histopathological scoring & hair follicle analysis

A 1 cm^2^ area of skin was excised from the right dorsal surface and fixed in 10% neutral buffered formalin for 24 h before processing and embedding in paraffin wax according to standard procedures. Vertical sectioning was chosen to allow for optimal detection of inflammatory cells whilst enabling follicular counts. 3 μm longitudinal sections were cut and stained with haematoxylin and eosin for histopathological analysis. Skin sections were scanned using an Aperio Scanscope. CD8 and NKG2D in situ hybridization (ISH) was carried out using the RNAScope technique^48^ according to the manufacturer's protocols. Briefly, tissue sections were deparaffinised and incubated with epitope retrieval solution 2 for 15 min at 88°C followed by ACD protease for 10 min before hybridization with probe Mm‐Klrk1 (ACD, Cat.No.430808) and probe Mm‐Cd8a‐C2 (ACD, Cat.No. 401688‐C2) or duplex negative control probes Mm‐DapB (ACD, Cat.No. 320758). Bound probe was visualized following amplification with RNAScope 2.5 LS Duplex Reagent Kit‐(RED/BROWN) (ACD Cat.No. 322440). Staining was visualized by light microscopy. Number of hair follicles and hair follicle width were analyzed using ImageJ software edition ImageJ2. Images were imported using the bio‐format importer plug in. The number of follicles were counted using the cell counter plugin. All follicles on all sections in the dermal fat layer were counted. Follicle width was calculated with use of the circle tool to capture follicle area and from it calculate the width. All follicles on all sections in the dermal fat layer were included in the width analysis.

#### 3.3.7 Flow cytometric analysis of mouse cutaneous lymph nodes and blood

Cutaneous lymph nodes and spleens from mice treated either orally or topically (treated and untreated side, respectively) were pooled and minced in DPBS/2% FBS/2 mM EDTA, filtered through 70 μM strainers and spun at 350 g for 5 min. Blood was collected in EDTA‐coated tubes and red blood cells were lysed using BD FACS lysing solution (as was red blood cells from spleen single cell suspensions). Cell differential were obtained from haematology analyzer Sysmex XE‐2100 (Sysmex Corporation, Japan). Equal cell numbers for each sample were incubated with anti‐mouse CD16/CD32 block to prevent unspecific binding and then stained for 30 min at 4°C with the following anti‐mouse antibodies (all from BD); FITC CD3e (145‐2C11), APC‐Cy7 TCRβ (H‐57‐597), BV510 or BV786 CD8α (53‐6.7), BV711 NKG2D (Cx5), BUV395 CD4 (GK1.5), BV711 or BV605 CD44 (IM7), BUV395 CD45 (30‐F11), PerCP‐Cy5.5 CD19 (1D3) and PE‐Cy7 CD62L (MEL‐14). For Ki‐67 (PE‐Cy7, B56) analysis, the cells were permabilised and fixed in eBioscience Fix/Perm buffer, followed by Ki‐67 antibody staining in eBioscience permeabilisation buffer for 1 h at room temperature. The cells were analyzed on a BD LSRII flow cytometer and analysis was performed with FlowJo (Tree Star) software.

#### 3.3.8 Mouse skin RNA preparation

RNA was prepared according to a standard phenol‐chloroform total RNA extraction method where skin samples were homogenized in QIAzol^®^ Lysis Reagent (QIAGEN Sciences, Maryland 20847, USA), added chloroform, vortexed and centrifuged for phase separation. The RNA containing phase was ethanol precipitated and purified on an Applied Biosystems 6100 Nucleic Acid Prep Station. RNA integrity was measured on the Fragment Analyzer platform (AATI, IA, USA) using standard sensitivity RNA analysis kit.

#### 3.3.9 Taqman analysis

cDNA was synthesized from mouse skin RNA using the High Capacity cDNA Reverse Transcription Kit from Life Technologies. Gene expression was performed on the QuantStudio™ 7 Flex Real‐Time PCR System (Applied Biosystems, Foster City, CA, USA) using custom made TaqMan^®^ Array Micro Fluidic Cards. Results were analyzed with the QuantStudio™ 7 Flex Real‐Time PCR System software. Detailed description in Supplementary materials.

### 3.4 In vitro experiments using human cell systems

Human Peripheral Blood Mononuclear Cells (PBMCs) were isolated by Sepmate technique (Stemcell Technologies) from heparinized venous blood from healthy volunteers. Primary human CD8^+^ T‐cells were further isolated using a CD8^+^ T cell Isolation Kit according to protocol (Miltenyi Biotec). Experiments were approved by the local ethical committee in Gothenburg (033‐11).

#### 3.4.1 In vitro stimulation of human cells

PBMCs and CD8^+^ T‐cells were stimulated in vitro with plate bound anti‐CD3 (OKT3) in complete media in the presence or absence of different JAK inhibitors; tofacitinib, ruxolitinib and a selective JAK1 inhibitor, Cmpd A (Table 1) at 3–3000 nM. Soluble anti‐CD28 were added to the CD8^+^ cultures to facilitate co‐stimulation. After 40–48 h, supernatants were harvested for mediator analysis and the cells were washed and resuspended in RLT buffer (Qiagen) for RNA isolation.

**TABLE 1:** In vitro potency and selectivity of JAK inhibitors

Compound	Structure	JAK1 IC_50_ (μM)^a^	JAK2 IC_50_ (μM)^a^	JAK3 IC_50_ (μM)^a^	TYK2 IC_50_ (μM)^a^	IL‐4, STAT6‐Luc IC_50_ (μM)^b^	IL‐13, STAT6‐Luc IC_50_ (μM)^b^	Solubility (μM)^c^
Tofacitinib	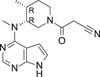	0.024	0.11	0.063	1.9	0.10	0.12	>1000
Ruxolitinib	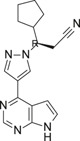	0.022	0.024	1.7	0.067	0.11	0.082	678
Cmpd A—Systemic	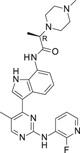	0.027	18	>30	NA	0.084	0.12	>337
Cmpd B—Local	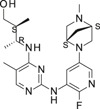	0.0093	>30	>30	NA	0.11	0.10	>955
Cmpd C—Local & systemic	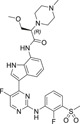	<0.003	1.2	>30	NA	0.024	0.034	6.6

^a^ Enzymes were assayed at a physiological ATP concentration of 5 mM.

^b^ Cellular (U937 monocytes) interleukin‐induced JAK‐STAT6‐luciferase assay.

^c^ Phosphate buffer, pH 7.4.

#### 3.4.2 Cytokine analysis in supernatants from human cell cultures

Analysis of human cytokine panels was carried out using a Luminex assay kit from Merck Millipore (Cat# HCYTMAG‐60K‐PX41) and Mesoscale Discovery (MSD) immunoassay kits (Cat# K211AEB‐2 and K15049D) as recommended in the respective supplier protocols.

#### 3.4.3 Human CD8^+^ T‐cell RNA preparation

RNA was isolated from 24 human CD8^+^ T‐cell samples exposed to Cmpd A, ruxolitinib or tofacitinib by means of RNeasy Mini kit with on‐column DNA digestion (QIAGEN, Hilden, Germany) and eluted in 50 μL Rnase‐free water. RNA integrity was measured on the Fragment Analyzer platform (AATI, IA, USA) using standard sensitivity RNA analysis kit.

#### 3.4.4 Next generation RNA sequencing (mouse and human)

The mouse RNA showed low RIN‐values and TruSeq Stranded Total RNA with RiboGold depletion (Illumina, CA, USA) were therefore chosen for library creation as it is less sensitive to low quality RNA than the TruSeq Stranded mRNA kit (Illumina, CA, USA). Mouse RNA was diluted to 50 ng/μL and used as input to create RNA libraries using TruSeq Stranded Total RNA with RiboGold depletion (Illumina, CA, USA) following standard instructions with the exception that no fragmentation of samples was made due to low RIN‐values. Human RNA was diluted to 5 ng/μL and used as input to create mRNA libraries using TruSeq Stranded mRNA kit (Illumina, CA, USA) following standard instructions. Libraries (both mouse and human) were validated on the Bioanalyser (Agilent, CA, USA) using DNA1000 chip and the concentration was determined using Quant‐iT dsDNA High Sensitivity assay kit on the Qubit fluorometer (Thermo Fisher, MA, USA). Sample libraries were pooled in equimolar concentrations and diluted and denatured according to Illumina guidelines. Sequencing was performed using a High Output 2 x 76 bp kit on an Illumina NextSeq500 to an average depth of 5.6 million (mouse) and 13.2 million (human) reads.

#### 3.4.5 RNAseq data processing and availability (mouse and human)

Base calling were done by bcl2fastq from Illumina. Reads were mapped to corresponding genome (mouse genome version mm10 or human genome version hg38) with HiSat2 (version 2.0.1‐beta (mouse) and 2.0.4 (human)), counts were generated using featurecounts (version 1.4.4). Normalized counts (from DESeq2) were used for all plotting, while counts were used for statistical testing. The datasets discussed in this publication have been deposited in NCBI's Gene Expression Omnibus and are accessible through GEO SuperSeries accession number GSE94237 (https://www.ncbi.nlm.nih.gov/geo/query/acc.cgi?acc=GSE94237).

The individual SubSeries are GSE94235 (human CD8^+^ T‐cells) (https://www.ncbi.nlm.nih.gov/geo/query/acc.cgi?acc=GSE94235) and GSE94236 (mouse skin samples) (https://www.ncbi.nlm.nih.gov/geo/query/acc.cgi?acc=GSE94236).

#### 3.4.6 ALADIN scores

IFN (Cxcl9, Cxcl10, Cxcl11, Stat1, and Mx1) and CTL (Cd8a, Gzmb, Icos, Prf1) ALADIN scores for the Taqman data were calculated for each group as described previously.^9^, ^49^ Briefly, z‐scores were calculated for each gene and sample relative to the mean and standard deviation of the healthy group. Signature scores are then calculated as the mean of the z‐scores within each group.

#### 3.4.7 STAT pathway genes

Identification of STAT pathway genes were done by Ingenuity® Pathway Analysis (Qiagen). Genes found downstream of STAT1‐6 were extracted for further processing. Genes found in two or more STAT pathways were excluded from further analysis, resulting in the following number of unique genes for each STAT pathway, STAT1: *n* = 235, STAT2: *n* = 38, STAT3: *n* = 582, STAT4: *n* = 221, STAT5A: *n* = 64, STAT5B: *n* = 187, STAT6: *n* = 164.

#### 3.4.8 RNAseq differential gene expression and enrichment analysis

Differential gene expression were tested using DESeq2 for mRNAseq data^50^ in R (version 3.3.0). Differentially expressed genes were defined using FDR <0.001 & |FC|>4 as cutoffs for the mouse data and FDR <0.05 & |FC|>2 for the human data. Network analysis were performed on differentially expressed genes using the “Compare Experiments Workflow” in Metacore™ (version 6.27, Thomson Reuters). Visualizations of Gene ontology analyses were done in Cytoscape (v3.6.0). Heatmaps for Taqman and mRNAseq results were created using heatmap.2 function from gplot in R (version 3.3.0). The mean expression of each group was used in the plots.

### 3.5 Statistical analysis

Statistical analyses have been performed using RStudio software (R version 3.2.3). Unpaired two‐sided Student's *t*‐test, one‐way analysis of variance (ANOVA) followed by Dunnett's two‐sided post hoc tests for multiple comparisons were used to analyze data as indicated. The number of hair follicles and mean follicle width showed violation of the assumption of homoscedasticity (equal variance across groups) which led to the use of a Welch ANOVA and following welch‐test for pairwise comparisons among groups. Cytokine data in Figure 6 show a right skewed distribution and was log‐transformed prior to performing linear regression on the dose‐response data. A linear model with donor, treatment and dose was fitted to the data and slopes for different treatment effects compared. Due to the small sample size, regular tests of normality have low power and was not performed. However, visual inspection of diagnostic plots after log‐transformation indicates a better fit to a log‐normal distribution. Data were considered significant if *p*‐values were below 0.05 or significantly different if 95% confidence intervals are not overlapping. Details of each statistical test, *n* and *p*‐values are reported in the results section, figure legends and/or supplementary tables.

## 4 RESULTS

### 4.1 Characteristics of selective JAK1 inhibitors and pan‐JAK inhibitors

To investigate the efficacy of JAK1 inhibition in AA, we selected three different tool compounds. The selection criteria included in vitro kinase (JAK) inhibition, cellular potency, solubility, physio‐chemical and in vivo pharmacokinetic properties. The compounds (designated Cmpd A,^40^ B (Supplementary materials) and C^42^) were compared to ruxolitinib^43^, ^51^ and tofacitinib^44^ in specific JAK inhibition assays (JAK1, JAK2, JAK3 and TYK2), While compounds A‐C displayed >600‐fold selectivity for JAK1 over JAK2 and JAK3, tofacitinib and ruxolitinib were found promiscuous at physiologically relevant conditions as previously reported (Table 1).^44^ The functional efficacy of JAK inhibition was determined by stimulating U937 cells with IL‐4 or IL‐13 and measuring STAT6 activation using a luciferase reporter system. Inhibition of IL‐4 and IL‐13‐induced STAT6 activation was similar for all compounds except for Cmpd C, which was approximately three to four fold more potent (Table 1). With regards to the pharmacokinetic properties of compounds A‐C, Cmpd A demonstrated good properties for oral delivery leading to similar total exposures in both plasma and skin, where plasma showed an increased free exposure due to an estimated 10‐fold higher unbound fraction in plasma compared to skin (the latter value estimated from the Gillette equation^52^, ^53^). Cmpd B exhibited beneficial properties for local (transdermal) delivery without spillover to the circulation with ratios for the estimated average free exposures over the average cell‐based potency (IC_50_) of 0.02‐fold in plasma and 1.9‐fold in skin, whereas Cmpd C had properties permissive for either systemic, including skin exposure, or solely local exposure (Table 2).

**TABLE 2:** In vitro and in vivo DMPK properties of Cmpd A, B & C

	In vitr*o* mouse & physical chemical properties					In vivo mouse pharmacokinetics			
Compound	LogD_7.4_	Basic pKa's	*f*_u_, _plasma_	*f*_u, tissue_ ^a^	Caco AtB 10^−6^ cm/s/Efflux ratio^b^	Dose i.v./p.o. (mg/kg)	CL (ml/min/kg)	*V*_ss_ (L/kg)	Oral % F
Cmpd A—Systemic	2.9	7.9	0.038	0.003	21/1.0	0.5/300	32	9.1	100
Cmpd B—Local	1.5	8.6 & 6.5	0.74	0.063	0.3/14	1.0/1.0	38	8.4	16
Cmpd C—Local & systemic	2.6	7.9	0.11	0.011	6.7/2.4	1.0/2.0	26	6.9	86

^a^ Obtained from the rearranged Gillette equation^29^ ^,^ ^30^: *f*_u, tissue_ = 1/(((*V*_ss_ – *V*_blood_)/*V*_tissue_)/*f*_u, plasma_), where *V*_blood_ = 0.07 L/kg and *V*_tissue_ = 0.6 L/kg and blood to plasma ratio was assumed equal to 1.

^b^ Efflux ratio = BtA/AtB.

### 4.2 Selective JAK1 inhibition is sufficient to restore hair growth and resolve inflammation

**FIGURE 1:**
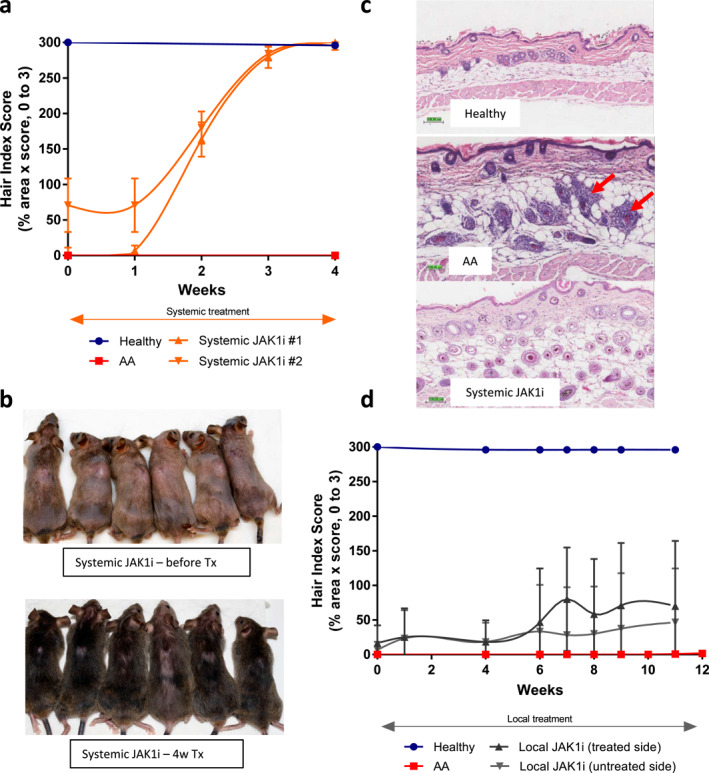
Complete restoration of hair growth and resolution of inflammation by systemic JAK1 inhibition. (a) Hair Index Score (Mean ± SD) demonstrating complete regrowth within 4 weeks of treatment with Cmpd A (orally for systemic exposure, denoted Systemic JAK1i) for two groups of mice (*n* = 6 per group, orange triangles up/down). Blue circles are healthy, age‐matched controls (*n* = 6) and red squares untreated alopecic mice (*n* = 6) monitored with no interventions. Individual scores in Table S1. (b) Photos taken before treatment (top) and after 4 weeks of systemic JAK1i (bottom) demonstrating hair regrowth. (c) H&E stains of skin sections from healthy, alopecic (AA) and treated mice (Systemic JAK1i with Cmpd A) demonstrate intense peri‐follicular inflammation (arrow), apoptosis and the induction of the anagen phase as opposed to the healthy telogen hair cycle phase (hair follicle in dermis within the AA group). (d) Average Hair Index Score (Mean ± SD) during 12 consecutive weeks of topical application of Cmpd B (treated side in grey triangles (up) and untreated side in grey triangles (down), *n* = 6 per group, denoted Local JAK1i). Bar denotes 100 μm, Tx = treatment

To investigate whether selective JAK1 inhibition alone was sufficient in restoring hair growth, mice with complete AA were given Cmpd A by daily servings for 4 weeks. The estimated average free exposure in plasma was 1.2‐fold IC_50_ by Cmpd A in contrast to the estimated average free exposure in skin which was 0.1‐fold IC_50_. Treatment induced a quick and complete restoration of hair growth and resolution of inflammation in the skin (Figure 1a,b, Table S1). Histopathological evaluation revealed that loss of fur in C3H/HeJ mice was associated with peri‐follicular lymphocytic inflammation, with an element of eosinophilia as well as apoptosis of follicular cells (Figure 1c). Systemic exposure to JAK1 inhibition for 4 weeks led to the resolution of local inflammation in all animals within the study (*n* = 6) as demonstrated by the lack of inflammatory cells in the skin sections (Figure 1c). Apoptotic cells remained apparent but decreased in incidence as compared to AA mice. The number of HFs in the anagen (growth) phase increased with the onset of disease and was not affected by 4 weeks of systemic JAK1 inhibition (Table S2). However, the mean width of the follicles in treated mice was slightly reduced as compared to follicles of untreated AA mice (Table S3).

### 4.3 Topical JAK1 inhibition fails to induce remission in mice with AA

To minimize the risk of side effects associated with systemic JAK inhibition, topical application may be a more clinically relevant route. In a previous study, pan‐JAK inhibitors appeared to be effective following local administration to the skin.^9^ We evaluated the exposure of 3% tofacitinib topically (0.1 g on the right side of the back) and found unbound skin exposures at IC_50_ (total concentrations of 465 μM, equalling to 23 nM free concentration using the Gillette equation) and 3–7‐fold IC_50_ in plasma (total of 216 ± 77 nM, ie 71–160 nM free) one hour after application. We could not confirm regrowth of fur using topical application of selective JAK1 inhibitors. Topical treatment for 12 weeks with Cmpd B did not restore hair growth despite giving a local free skin exposure of 1.9‐fold IC_50_ (Figure 1d). A negligible average free plasma exposure was detected (0.02‐fold IC_50_). These findings were confirmed by Cmpd C which combines good oral bioavailability with significant tissue retention. Here, systemic exposure (with estimated average free concentrations in plasma of 1.4‐fold IC_50_ and similarly for skin at 0.5‐fold IC_50_) induced hair growth and resolved inflammation, whereas topical application (with an insignificant coverage of 0.02‐fold IC_50_ in plasma and 0.43‐fold IC_50_ in skin) did not. Collectively, these data demonstrate that systemic JAK1 inhibition is needed for efficacy.

### 4.4 Systemic JAK1 inhibition markedly reduces the number of pathogenic NKG2D^+^ CD8^+^ T cells

**FIGURE 2:**
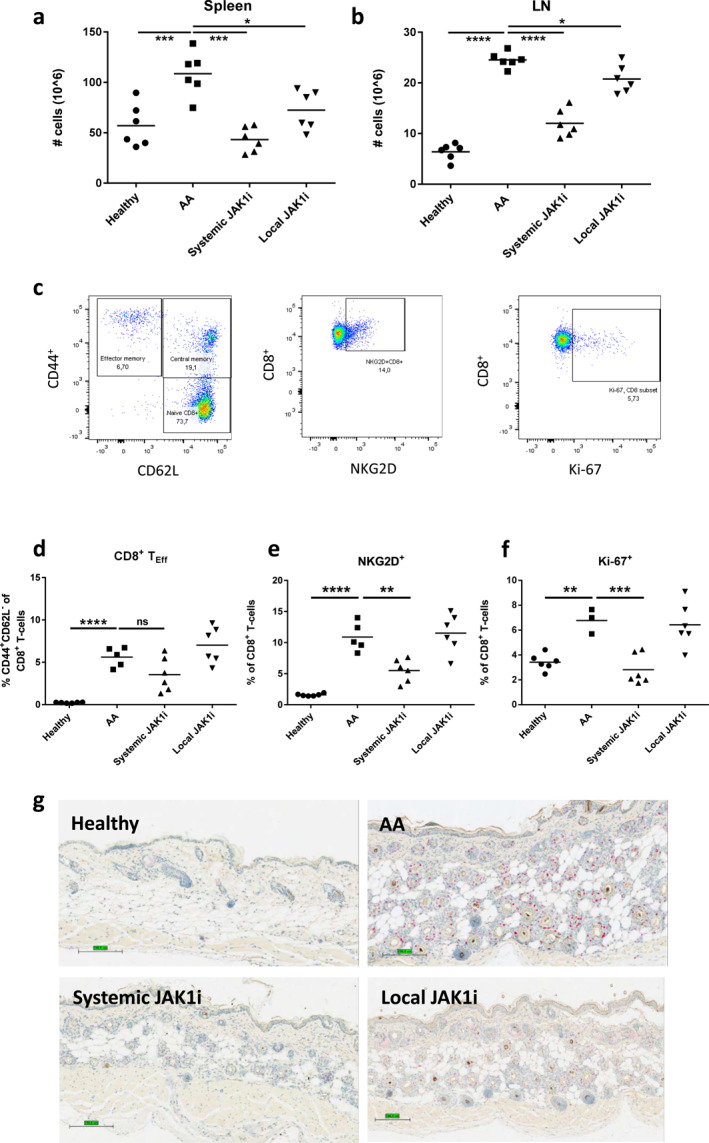
Systemic, but not local, JAK1 inhibition reduces LN hypercellularity as well as the number of CD8^+^ T_Eff_ cells in LNs and skin. (a) Hypercellularity in spleen and (b) skin draining lymph nodes and effects of JAK1 inhibition (****p‐value AA vs. systemic JAK1i and AA vs. Healthy <0.0001, *p‐value AA vs. local JAK1i = 0.022). (c) Gating strategy (Live, CD3^+^TCRb^+^CD8a^+^). Effect of JAK1i inhibition in skin draining lymph nodes (d) on the effector CD8^+^ T‐cells (T_Eff_, ****p‐value AA vs. Healthy <0.0001), (e) NKG2D^+^ expression on CD8^+^ T‐cells with and without JAK1 inhibition (**p‐value AA vs. systemic JAK1i = 0.0015 and ****p‐value AA vs. Healthy <0.0001), and (f) expression of the proliferation marker Ki‐67 on CD8^+^ T‐cells (***p‐value AA vs. systemic JAK1i = 0.0010, and **p‐value AA vs. Healthy = 0.0042). Statistical analysis (1‐way ANOVA and Dunnett's post hoc tests). (g) Localization of NKG2D^+^ and CD8^+^ cells in the skin by means of RNAScope, NKG2D^+^ (brown) and CD8^+^ (red).

Although the clinical manifestation of AA is local, there are signs of systemic inflammation. Pathogenic NKG2D^+^ CD8^+^ T cells are not only found in the skin, but also in skin draining lymph nodes (LNs). Skin draining LNs as well as the spleen display marked hypercellularity.^9^ We assessed the impact of systemic or local JAK1 inhibition on hypercellularity in spleen and LNs and evaluated the presence of pathogenic CD8^+^ T cells in LNs and skin. Following systemic JAK1 inhibition with Cmpd C the hypercellularity of both skin draining LNs (axillary and inguinal) and spleen was drastically reduced, whereas it was only marginally affected by topical treatment (Figure 2a,b). Although small, this reduction could indicate some minor spillover from local JAK1 treatment into the systemic circulation.

The frequency of the pathogenic NKG2D^+^ CD8^+^ T cell subpopulation in skin draining LNs was significantly reduced following systemic, but not local JAK1 inhibition. A smaller reduction in the total frequency of CD8^+^ effector cells was also observed (Figure 2c–e). Moreover, proliferation of the CD8^+^ T‐cells in AA mice, as measured by intracellular staining for Ki‐67, was significantly decreased by systemic JAK1 inhibition (Figure 2f). Importantly, the expression of NKG2D and the number of CD8^+^ T‐cells in the skin was also markedly reduced following systemic JAK1 inhibition, as visualized using RNAScope technology (Figure 2g). Although there appeared to be a slight reduction in the number of CD8^+^ T cells in the skin of mice treated topically, it was not complete and not comparable to the substantial reduction as seen with systemic treatment (Figure 2g).

### 4.5 4 weeks of JAK1 inhibition failed to induce treatment free remission in AA mice

**FIGURE 3:**
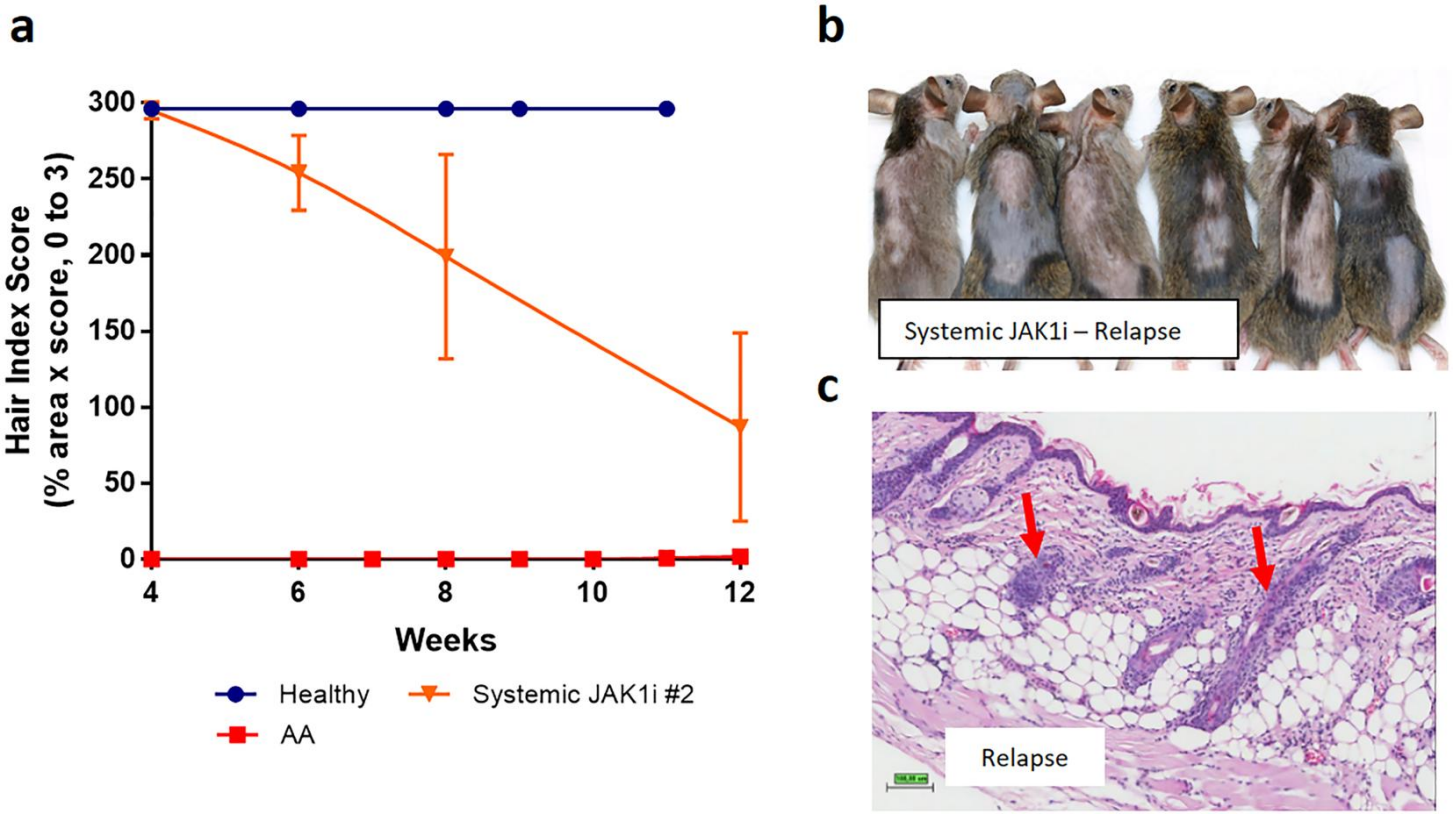
Complete hair restoration does not secure post treatment remission. (a) Hair Index Score (Mean ± SD) demonstrating AA relapse immediately upon cessation of the 4 weeks long JAK1i treatment (Cmpd A) followed for 8 weeks, from week 4–12 (orange triangles (down), *n* = 6). Blue circles are healthy, age‐matched controls (*n* = 6) and red squares untreated alopecic mice (*n* = 6) monitored with no interventions. Individual scores in Table S1. (b) Photo taken 8 weeks after cessation of systemic JAK1i demonstrating hair loss during relapse. (c) H&E stains of skin section from relapsing mice after 8 weeks off treatment demonstrate intense peri‐follicular inflammation (arrow), apoptosis and the induction of the anagen phase as opposed to the healthy telogen hair cycle phase in Figure 1 (hair follicle in dermis within the 8w off treatment group). Bar denotes 100 μm.

It's been previously shown that 12 weeks of ruxolitinib or tofacitinib treatment induced stable remission during a 3 months follow up.^9^ An alternative approach to reduce the risk of side effects is to minimize the duration of treatment. We therefore asked whether mice treated for 4 weeks with JAK1 inhibitors (Cmpd A) would remain in remission after treatment was halted. Despite complete restoration of hair growth after 4 weeks of treatment, the mice relapsed after treatment cessation and after 8 weeks their hair score index and levels of peri‐follicular inflammation had returned to that seen in untreated mice (Figure 3a–c). Interestingly, the number of anagen follicles was returned to normal (Table S2). We speculate that the failure to remain in remission following 4 weeks of treatment may be due to the systemic persistence of pathogenic NKG2D^+^CD8^+^ memory T cells. Although CD8^+^ T effector cells appeared to be absent from the skin following JAK1 inhibition, there was still a significant, albeit reduced, population in the skin draining LNs (Figure 2d,e). Furthermore, treated mice also displayed elevated levels of IFN‐γ in the circulation as compared to healthy controls, indicating a sustained systemic dysregulation (Table S4).

### 4.6 JAK1 inhibition normalizes the skin transcriptome of AA mice

**FIGURE 4:**
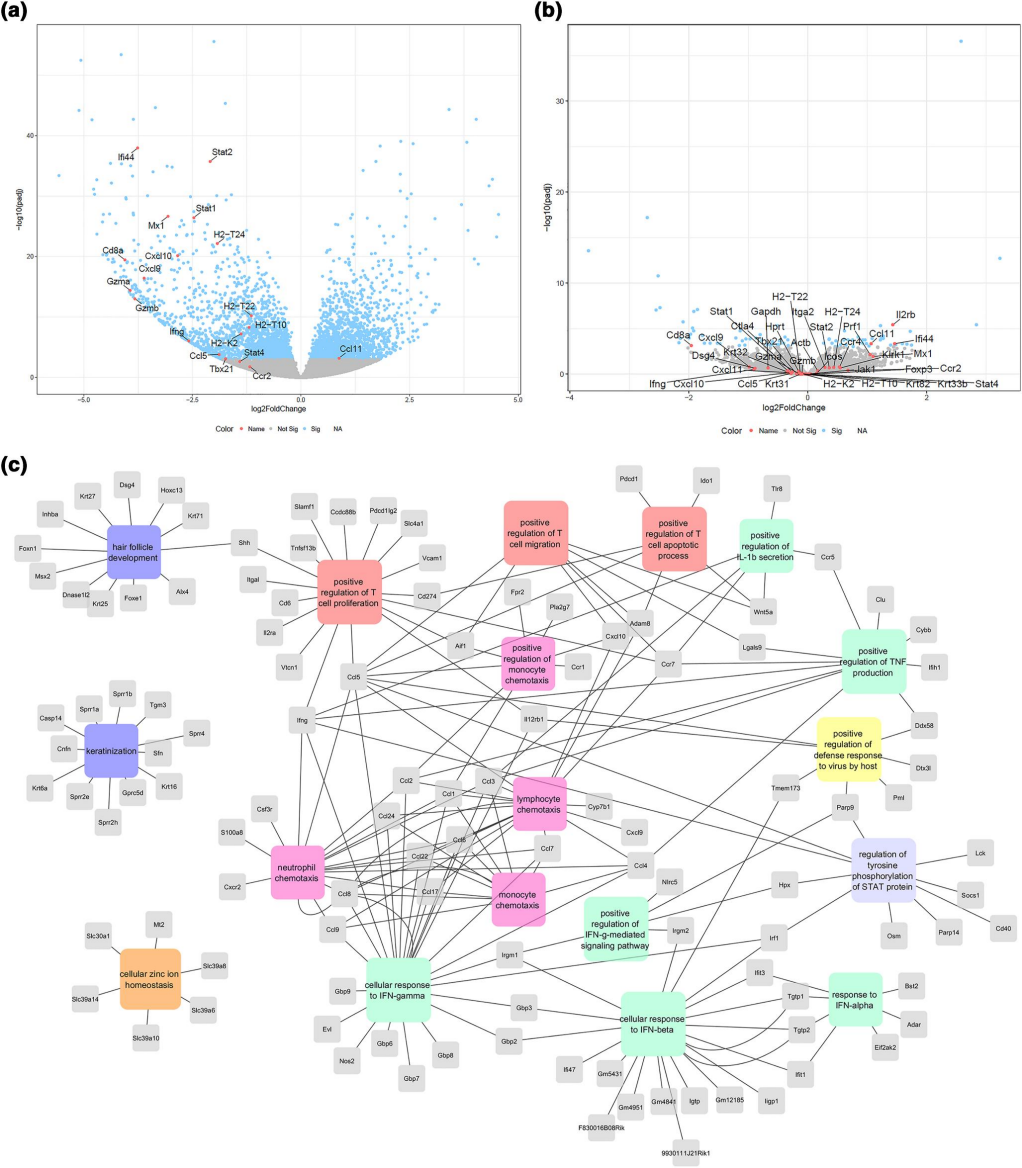
Gene expression in vivo. (a) Volcano plot of gene differentials between AA and healthy controls, highlighting some of the most important genes in disease. (b) Volcano plot for systemic JAK1i versus healthy. (c) Gene ontology analysis results showing important pathways involved in AA being activated in AA mice as compared to HC mice (DEG cutoff FDR <0.001 & log2FC < −1). Selected processes are shown with connecting genes.

**FIGURE 5:**
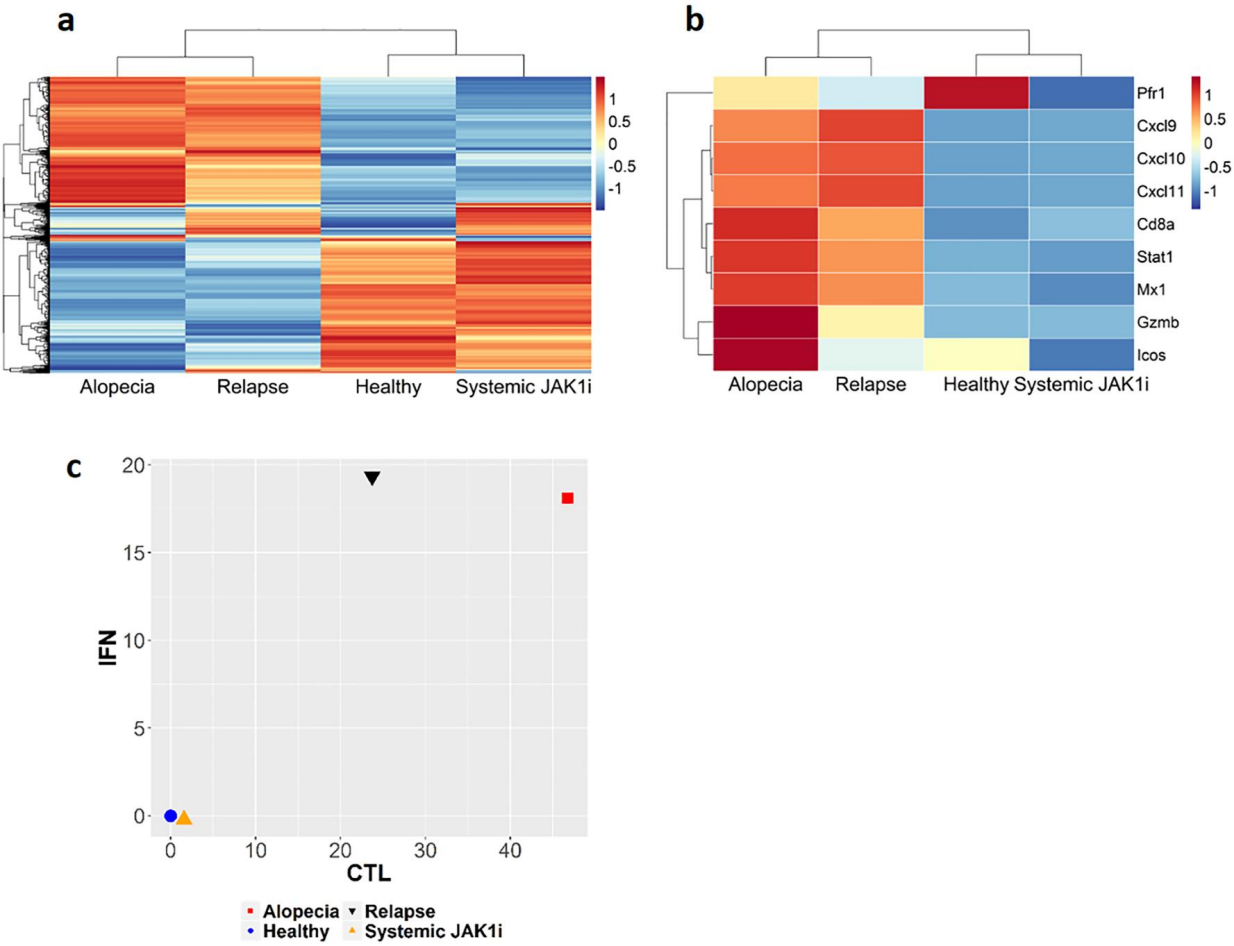
JAK1 inhibition normalizes gene expression. (a) Heatmap of top 10% most variable genes (based on inter‐quartile range of normalized counts, *n* = 3900) from RNA seq. (b) qPCR analysis of a selection of interferon genes (Cxcl9, Cxcl10, Cxcl11, Stat1, Mx1) and CTL genes (Cd8a, Gzmb, Icos, Pfr1) performed on skin samples and presented as a heatmap of relative mean expression per group. (c) The selected IFN and CTL genes from (b) were used to calculate the ALADIN score, presented as a PSA plot.

To evaluate whether the reversal of hair loss induced by systemic JAK1 inhibition was also reflected at the transcriptional level, we performed RNAseq on skin samples from healthy mice, AA mice, AA mice in remission that had been treated with a JAK1 inhibitor and treated AA mice that had relapsed following treatment cessation. As expected, we found a striking difference in the expression profile comparing healthy and AA mice (Figure 4, Table S5). Up‐regulated genes in AA mice as compared to healthy included Th1/CD8^+^ T cell‐associated genes such as Ifng, Stat1, Stat2, Stat4, Gzma, Gzmb, Cxcl9, Cxcl10, Cxcl11 and Tbx21, but also genes indicating an impaired immune privilege such as the MHC I genes *H2‐t24*, *H2‐t22*, *H2‐t10* and *H2‐k2* (Figure 4a). Most of these were normalized with selective JAK1 inhibition (Figure 4b). Gene enrichment analysis for biological processes on the up‐regulated genes in AA mice revealed significantly overrepresented immune signatures including inflammatory responses, chemotaxis of leucocytes, response to IFN‐γ, defence against virus and T cell proliferation (Figure 4c). But also, a significant suppression in processes specific to HFs such as the hair cycle and keratinocyte differentiation (Figure 4c). There was a striking similarity between AA mice and mice that had relapsed following JAK1 inhibition and importantly, also when comparing AA mice in remission following JAK1 treatment versus healthy mice (Figure 5). Thus, it is evident that the systemic JAK1 inhibition brings the expression of most genes back to healthy levels (Figure 5). The RNAseq data was validated using qPCR for selected genes involved in Th1 immunity and cytotoxicity, a number of JAKs, and the downstream responder genes STATs and SOCS (Figure 5b, Figures S1 and S2). Calculation of Alopecia Areata Disease Activity Index (ALADIN) scores^9^ further validated the similarity between healthy and JAK1‐treated mice in remission as well as AA mice and mice that relapsed following cessation of treatment, respectively (Figure 5c).

### 4.7 Selective JAK1 inhibition is sufficient to dampen IFN‐γ release from human CD8^+^ T cells

**FIGURE 6:**
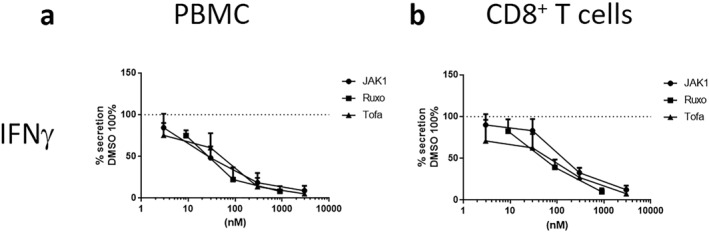
Selective JAK1 inhibition demonstrates overall similar immunosuppressive responses as panJAK inhibition. (a) The levels of secreted IFN‐γ from plate bound anti‐CD3‐stimulated PBMCs (*n* = 8) and (b) IFN‐γ from CD8^+^ T‐cells (*n* = 8) are not significantly different between the different JAK inhibitors (JAK1 = Cmpd A, Ruxo = Ruxolitinib, Tofa = Tofacitinib). Supernatants were collected after 40–48h of stimulation and secreted mediators analyzed (shown in Figure S3). DMSO control was set to 100%. Each data point depict Mean  +  SEM. Statistical analysis was performed by means of linear regression analysis.

Next, we investigated whether selective JAK1 inhibition had a comparable effect to pan‐JAK inhibition in human cells from healthy volunteers. We assessed cytokine secretion from PBMCs, or isolated CD8^+^ T cells, following their activation using anti‐CD3/anti‐CD28. Importantly, the production of IFN‐γ, considered a main driver of AA, was inhibited in a dose‐dependent manner and at an equivalent efficacy to the pan‐JAK inhibitors tofacitinib and ruxolitinib in both PBMCs as well as isolated CD8^+^ T cells (Figure 6a,b).^54^ Furthermore, the production of IL‐6, IL‐10, TNFβ, IL‐1β and IL‐4 by activated PBMCs were similarly inhibited by all three JAK inhibitors (Figure S3). Interestingly, we observed differences between selective and non‐selective JAK1 inhibition with regards to the IL‐1 receptor antagonist (IL‐1RA), TNFα, IL‐17, CCL4/MIP‐1β and CXCL10/IP‐10, demonstrating contrasting JAK‐dependence for the production of different cytokines (Figure S3). These results, in conjunction with the in vivo data, indicate that inhibiting JAK2, JAK3 and TYK2 activity is not required for efficacy in treating AA and could thus be avoided to minimize the adverse effect risk profile that has been linked to the inhibition of these kinases.

### 4.8 The transcriptome of activated human CD8^+^ T cells is affected to a similar degree by selective JAK1‐ and pan‐JAK inhibition

**FIGURE 7:**
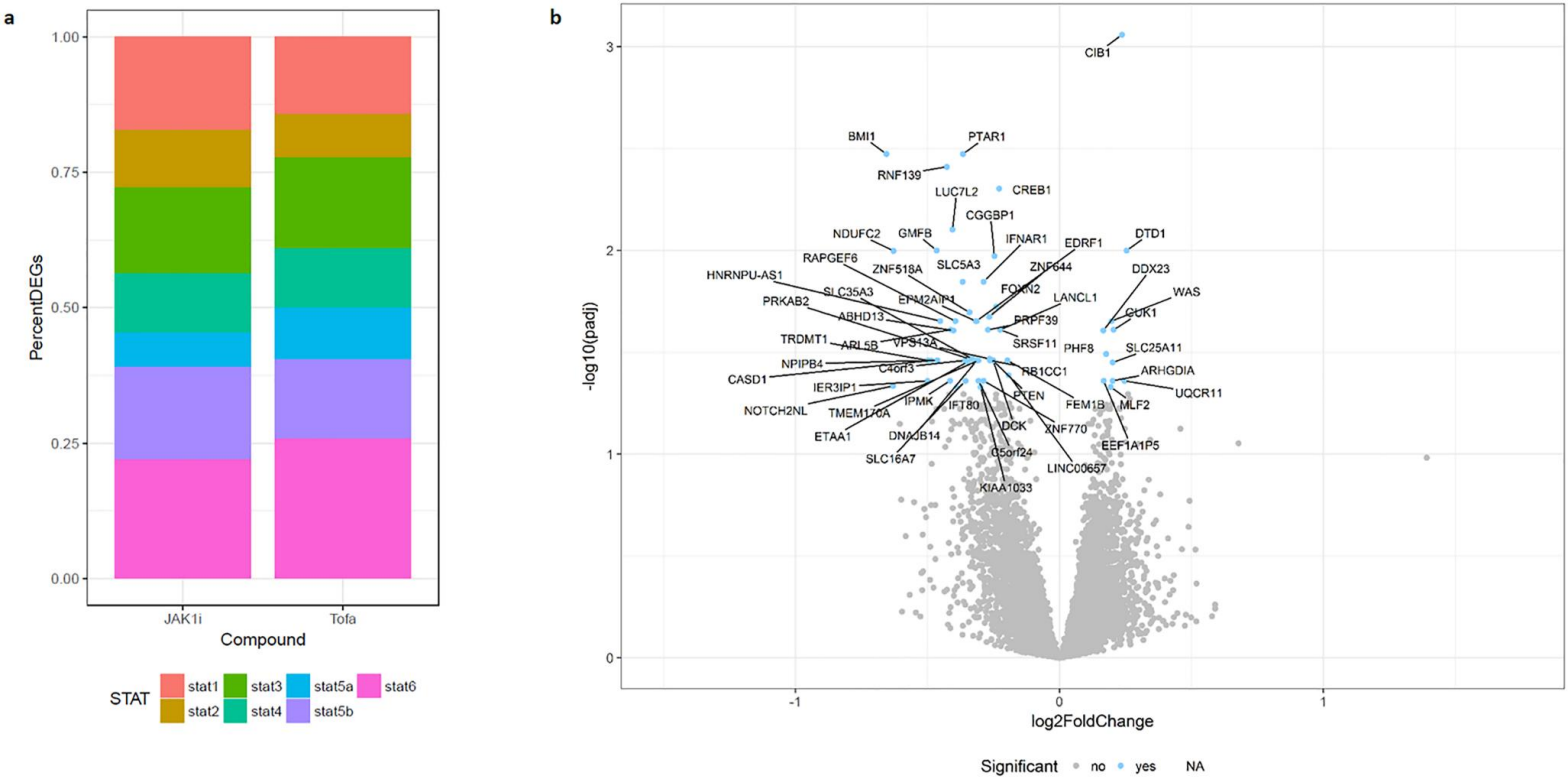
Selective JAK1 inhibition affects similar genes downstream of STAT1‐6 to Tofacitinib, with the exception of STAT5a. (a) The percent of genes uniquely found downstream of each STAT that are being differentially expressed in human CD8^+^ cells stimulated with αCD3/αCD28 for 40–48h and treated with Cmpd A, a selective JAK1 inhibitor (JAK1i) or Tofacitinib (Tofa) at concentrations giving equivalent inhibition of IFN‐γ release. The number of unique genes evaluated downstream of each STAT are: STAT1: *n* = 235, STAT2: *n* = 38, STAT3: *n* = 582, STAT4: *n* = 221, STAT5A: *n* = 64, STAT5B: *n* = 187, STAT6: *n* = 164. (b) Volcano plot demonstrating the few differentially expressed genes with selective JAK1 versus pan‐JAK inhibition (Tofacitinib).

To get a more complete picture of the impact of specific JAK1 versus pan‐JAK inhibition on activated human CD8^+^ T cells, we used RNAseq to assess differences in gene expression profiles induced by the different compounds. When comparing the number of regulated genes downstream of STAT proteins between cells treated with a JAK1 inhibitor and tofacitinib, a similar pattern emerged (Figure 7a). Although tofacitinib appeared to have a slightly stronger effect, there were few differentially regulated genes between the two compounds (all with a less than 2‐fold difference), further supporting the hypothesis that selective JAK1 inhibition will be as efficacious as pan‐JAK inhibition in AA (Figure 7b). Data is not shown for ruxolitinib as in vitro concentrations were not equipotent to tofacitinib and Cmpd C.

## 5 DISCUSSION

The results of this study indicate that selective JAK1 inhibition will be as efficacious as pan‐JAK inhibitors in resolving inflammation and restoring hair growth in patients with Alopecia Areata (AA), with a reduced risk of causing respiratory infections, anaemia, neutropenia and thrombocytopaenia as has been associated with some pan‐JAK inhibitors.^34^, ^35^, ^36^, ^37^, ^39^ Importantly, we have shown that systemic exposure is essential for efficacy and that treatment likely needs to be extended beyond that of restored hair growth to block relapse due to the presence of residual memory cells in secondary lymphoid organs. For this reason, we further speculate that topical application is unlikely to induce treatment‐free remission unless local application also results in systemic exposure.

It is now well established that JAK inhibition is efficacious in a broad range of diseases from oncology to inflammatory disorders and potentially also in allergic conditions, such as asthma and atopic dermatitis.^55^, ^56^, ^57^, ^58^, ^59^, ^60^ Recently, several case reports and small investigative clinical studies have demonstrated impressive efficacy of JAK inhibition in AA, using for example, tofacitinib (Xeljanz^®^ launched for rheumatoid arthritis), ruxolitinib (Jakavi^®^/Jakafi^®^ launched for myeolofibrosis),^9^, ^18^, ^19^, ^20^, ^21^, ^22^, ^23^, ^24^, ^25^, ^26^, ^27^, ^28^, ^29^ and recently also baricitinib (Olumiant^®^ launched for RA), Atopic Dermatitis and now also for AA and vitiligo (as Opzelura)^29^, ^31^ (Press release https://investor.lilly.com/node/44896/pdf) and ritlecitinib (previously PF 6651600, in development for AA, Crohn's disease; RA, Ulcerative colitis, Vitiligo)^32^ (Press release https://www.pfizer.com/news/press-release/press-release-detail/pfizer-announces-positive-top-line-results-phase-2b3-trial). The beneficial effects are clearly dose‐ and concentration‐dependent with a narrow safety window regarding susceptibility to infections as well as anaemia, limiting their potential for non‐debilitating autoimmune or mild inflammatory conditions like AA.^34^, ^35^, ^36^, ^39^ Separating beneficial effects from side effects would widen the potential for JAK inhibitors also in milder inflammatory conditions as discussed by Yan et al.^38^ and Lensing and Jabbari.^37^ Here, we demonstrate that selective inhibition of JAK1 in AA show similar efficacy to pan‐JAK inhibitors. Furthermore, our data indicates that free circulating concentrations of just above the IC_50_ (concentration to achieve 50% inhibition) for a JAK1 inhibitor is enough for fast and complete restoration of hair growth as well as resolution of inflammation.

We observed similar effects on the cytotoxic lymphocyte (CTL) and IFN gene signature in the skin of JAK1‐treated mice as was previously reported for the pan‐JAK inhibitors tofacitinib and ruxolitinib.^9^ However, 4 weeks of JAK1 inhibition did not lead to treatment‐free remission, as compared to the 12‐week long period in the Xing et al. study, where no relapse was observed over a 3 months follow up period.^9^ This could potentially be due to different properties of the compounds used in the two studies. Unfortunately, the exposure of tofacitinib and ruxolitinib in the previous study was not reported.^9^ However, the similarity between the studies in terms of hair regrowth kinetics, combined with the normalization of the skin transcriptome following treatment, indicate that the relapse following 4 weeks of treatment was not a consequence of the pharmacology of the compounds. Instead, we believe that the longer treatment protocol used by Xing et al. is a more likely explanation. Indeed, although local skin inflammation was reversed following 4 weeks treatment, there were still signs of systemic dysregulation. For example, we observed a remaining, although significantly diminished, population of NKG2D^+^CD8^+^ T cells in skin draining LNs that could potentially act as a source of new pathogenic effector cells that could re‐populate the skin. Also, serum levels of IFN‐γ were elevated in AA mice, and levels were not restored by treatment, further suggesting an incomplete suppression of systemic inflammation. Thus, it is important to consider treatment effects not only on a local level, but also to assess whether systemic homoeostasis has been re‐established before treatment is halted. 4 weeks of treatment was clearly sufficient to abolish pathogenic effector CD8^+^ T cells in the skin, allowing for hair regrowth, but at the same time it was not sufficient to completely eliminate memory cells present in secondary lymphoid organs. This disparity could perhaps be a result of differential requirements for survival signals between CD8^+^ T cells residing at the two sites. Long term survival of memory CD8^+^ T cells is dependent on IL‐15 and IL‐7, both of which signal via JAK1/JAK3 and STAT5 to induce anti‐apoptotic molecules such as Bcl‐2 or BclX_L_.^61^, ^62^, ^63^ Blocking IL‐15RB has been shown to be efficient in preventing the development of AA and both IL‐15 and IL‐7 are expressed by cells in the HFs.^64^ In addition, blocking IL‐2, also signalling via JAK1/JAK3 and STAT5, has been shown to prevent hair loss equally well, however it is unclear as to whether this was due to reduced de novo effector cell generation/expansion as opposed to memory cell survival.^9^ Given that IL‐15, IL‐7 and IL‐2 all signal via JAK1, we cannot draw any conclusions regarding their respective importance in this model based on our findings using JAK1 inhibitors. Nevertheless, there are reports suggesting that IL‐2/IL‐15 signal strength, via their common receptor CD122, orchestrates effector versus memory development. Stronger signals are required for effector cell development whereas weaker signals are sufficient for sustaining the survival of memory cells.^65^ This provides an attractive explanation for the fact that the effector cells in the skin where quickly abolished by JAK1 inhibition whereas some memory cells in skin draining LNs remained after 4 weeks of treatment. Perhaps this can also explain the reported relapses upon treatment cessation in the clinic.^24^, ^25^

In addition to IL‐2 and IL‐15, blocking IFN‐γ can also prevent the development of AA in the C3H/HeJ model.^9^ IFN‐γ is believed to have a central role in abolishing the immune privilege of HFs and IFN‐γ produced by CD8^+^ effector T cells in the skin was shown to drive a “pathogen alert state” which included the up‐regulation of MHC I molecules.^66^ Also, mice injected with IFN‐γ have been shown to develop AA, further demonstrating its importance in driving the disease, although these findings were disputed in a follow‐up study.^67^, ^68^ Given the JAK1/JAK2 dependence of IFN‐γ receptor signalling this could be an important mechanism for the treatment of AA using JAK inhibitors. Whereas genes related to immune privilege appeared to be normalized following 4 weeks of JAK1 treatment, it would be reasonable to assume that this could quickly be reversed if remaining memory cells can re‐populate the skin following treatment cessation and yet again expose HFs to IFN‐γ.

Recent results using selective JAK1, JAK2 or JAK3 inhibitors, claim that JAK3 inhibition is equally effective to JAK1 inhibition in restoring hair growth in mice whereas JAK2 inhibition does not, indicating that the common γ‐cytokine signalling pathway is the primary therapeutic target in AA and that concomitant IFN‐γ inhibition is not necessary.^33^, ^69^ In support of these preclinical findings, the selective JAK3/TEC inhibitor, ritlecitinib, showed impressive efficacy and safety in a phase II clinical trial^32^, ^33^ which was confirmed in the phase III ALLEGRO trial (Press release https://www.pfizer.com/news/press-release/press-release-detail/pfizer-announces-positive-top-line-results-phase-2b3-trial), however, the JAK1/2/TYK2 inhibitor brepocitinib^70^ (PF‐06700841) was more effective (and less safe), with the caveat that only one dose per asset was tested in the phase II study. Further, previously published data showed that topical treatment is sufficient for good efficacy.^9^ There are however caveats with these conclusions as free fraction of drug in vivo (both locally in the skin and systemically) are not provided. Furthermore, selectivity ratios of the termed JAK1 and JAK2 selective inhibitors used are subtle and in some cases not determined at physiological relevant conditions, which makes the interpretation of results challenging.^71^, ^72^, ^73^, ^74^, ^75^ A limitation of our study was that we did not evaluate drug exposure at the epidermis/dermis level but at bulk concentrations with modelled free fractions of drug in the skin. However, there were similar levels of JAK1 inhibition in the skin when hair growth was initiated by systemic exposure and 4 to 19‐fold higher JAK1 inhibition from topical application when hair growth was not initiated, indicating that it was not the local JAK inhibition that induced the effects obtained. Naturally, there is no information on skin drug concentrations from published clinical case studies and there is to our knowledge not yet any randomized‐controlled clinical trials that potentially would reveal systemic versus skin exposures of the drug used. Given the outcome of the prospective analysis of 15 clinical trials by Yan et al^38^ that show significant and clinically promising efficacy and safety profiles for selective JAK inhibitors with different profiles, it seems obvious that interfering with JAKs independent of specificity will broadly inhibit signalling by many cytokines of importance in the manifestation of AA. Furthermore, demonstrates that also in clinical trials, systemic exposure to JAK inhibition is warranted for efficacy, supported by our pre‐clinical results. In contrast to Xing et al. and Dai et al., we could not induce any significant regrowth using topical JAK1 inhibition. Since levels of systemic exposure were not reported in the previous two studies, it is difficult to say whether the difference is a result of different pharmacokinetic properties of the compounds, but in an internal evaluation of 3% tofacitinib cream topically we found 5‐fold IC_50_ unbound concentrations in the circulation and free concentrations in the skin at the level of IC_50_. The JAK1 inhibitors used topically in the present study did appear to have a small but insufficient effect locally, but not systemically, as was expected given the minimal systemic exposure. However, Xing et al reported reduced NKG2D^+^ T cells not only in the skin but also in the LNs which indicates systemic exposure also in their study. Although hair regrowth was only reported on the dorsal back and not on the untreated abdominal side, there are significant differences between these sites in terms of hair loss. The abdominal side is generally the first to lose hair and also last to regain it after treatment (unpublished observation), this could potentially skew the results following topical administration. However, Dai et al. did report restored hair growth from topical administration without effects on numbers and frequencies of total CD8^+^ T cells, CD8^+^NKG2D^+^ T cells, and CD8^+^ T_E/M_ cells in skin‐draining LN cells, indicating no systemic effect albeit systemic exposure and effects were not investigated. Regardless of the cause behind the differential outcome between the studies, our data suggest that it is important not only to inhibit the local immune cells in the skin, but also to abolish the systemic memory cells to reach treatment‐free remission. Indeed, this conclusion is supported by a recent case study where local administration of ruxolitinib failed to induce hair regrowth in a patient with AU.^76^ Another case study reported some regrowth of hair using topical ruxolitinib, but there was also a concomitant reduction of circulating white blood cells, indicating systemic exposure.^21^

An interesting effect of JAK inhibition is that it seems to promote hair growth by initiating the anagen phase in normal C57Bl6 mice.^77^ This could be an additional reason for the impressive efficacy seen by JAK inhibitors in the treatment of AA, as triggering the induction of the hair cycle is likely another important factor to restore hair growth besides the inhibition of pathogenic immune cells. Perhaps this is the reason why most HFs were in the anagen phase after JAK1 treatment, similar to what was seen in untreated AA mice, but not in healthy mice.^77^

With regards to the cytokine release from stimulated human PBMCs and CD8^+^ T‐cells, selective JAK1 inhibition versus pan‐JAK inhibition showed an equivalent reduction of most of the cytokines examined. Importantly, IFN‐γ release from isolated CD8^+^ cells, as well as PBMCs, was significantly inhibited by all JAK inhibitors tested. Furthermore, we also observed inhibition of CXCL10 (IP‐10), which is up‐regulated in AA lesions and attracts pathogenic T cells via CXCR3.^78^ Interestingly, we observed some differences between the compounds specifically with regards to the IL‐1 receptor antagonist (IL‐1RA), TNFα, IL‐17 and CCL4. Polymorphisms in the IL‐1RA gene/‐s have been linked to an increased predisposition to the more severe forms of AA; alopecia totalis and universalis,^79^, ^80^ indicating a reduced ability to control IL‐1α and IL‐1β signalling. Both ruxolitinib and tofacitinib dramatically increased the release of IL‐1RA from PBMCs during stimulation whereas selective JAK1 inhibition did not. However, the release of IL‐1β was similarly and effectively inhibited by all JAK inhibitors, indicating that inhibiting JAK1 can be sufficient in this regard. In contrast, TNFα release was significantly less affected by JAK1 inhibition as compared to tofacitinib and ruxolitinib, possibly indicating that JAK1 inhibitors may not be as efficacious in patients with diseases such as rheumatoid arthritis and psoriasis, where anti‐TNFα treatment has proved successful. Nevertheless, JAK1 inhibition may work well in non‐responding patients, and indeed, this is the expected patient stratification group for orally available JAK1 inhibitors. IL‐17 was also not inhibited by JAK1 inhibition as opposed to the pan‐JAK inhibitors, perhaps questioning its efficacy in diseases such as psoriasis and ankylosing spondylitis where both pan‐JAK inhibitors and anti‐IL‐17 biologicals have shown great clinical efficacy.^81^ As for pan‐JAK inhibitors this was observed at very high, and most likely not clinically feasible doses, which may explain why the safety margins for tofacitinib in the treatment of psoriasis was not sufficient for approval by the food and drug administration. IL‐23 is critical in maintaining Th17/IL‐17‐producing cells as it signals via JAK2 and is therefore not inhibited by selective JAK1 inhibition, potentially explaining the lack of effect on IL‐17 levels.^82^ In terms of the effect on the transcriptional profile of activated CD8^+^ T cells, the impact of JAK1 inhibition was very similar to tofacitinib. This is not surprising as autocrine release of IL‐2, which signals via JAK1, is probably the most important factor resulting from anti‐CD3/anti‐CD28 stimulation of isolated T cells in vitro.^83^ This is in contrast to when a mixed population of PBMCs where stimulated and analyzed for cytokine release. In this setting, a more complex cytokine milieu and a wide variety of responder cells naturally resulted in some differentiation between specific JAK1 and pan‐JAK inhibition.

In conclusion, our data reveal that selective JAK1 inhibitors are likely to be as efficacious as pan‐JAK inhibitors in the treatment of AA, while reducing the risk of adverse events. Furthermore, we have demonstrated that prolonged systemic exposure is likely to be required for sustained efficacy and that local JAK inhibition is not sufficient to restore hair growth, unless it also results in systemic inhibition.

## CONFLICT OF INTEREST

All authors are or were at the time employed by AstraZeneca.

## AUTHOR CONTRIBUTIONS

**Johan Mattsson**: Conceptualization (Lead); Data curation (Equal); Formal analysis (Equal); Investigation (Equal); Methodology (Equal); Visualization (Equal); Writing – original draft (Lead); Writing – review & editing (Equal). **Elisabeth Israelsson**: Conceptualization (Equal); Data curation (Equal); Formal analysis (Equal); Visualization (Equal); Writing – original draft (Equal); Writing – review & editing (Equal). **Karin Björhall**: Conceptualization (Equal); Data curation (Equal); Writing – review & editing (Equal). **Linda Fahlén Yrlid**: Conceptualization (Equal); Data curation (Equal); Formal analysis (Equal); Visualization (Equal); Writing – original draft (Equal); Writing – review & editing (Equal). **Kristoffer Thörn**: Conceptualization (Equal); Data curation (Equal); Formal analysis (Equal); Writing – review & editing (Equal). **Anna Thorén**: Methodology (Equal); Writing – review & editing (Equal). **Emelie Andersén Toledo**: Methodology (Equal); Writing – review & editing (Equal). **Lisa Jinton**: Conceptualization (Equal); Data curation (Equal); Methodology (Equal); Writing – original draft (Equal); Writing – review & editing (Equal). **Lisa Öberg**: Conceptualization (Equal); Data curation (Equal); Formal analysis (Equal); Visualization (Equal); Writing – review & editing (Equal). **Cecilia Wingren**: Conceptualization (Equal); Data curation (Equal); Formal analysis (Equal); Methodology (Equal); Visualization (Equal); Writing – original draft (Equal); Writing – review & editing (Equal). **Sofia Tapani**: Formal analysis (Lead); Writing – review & editing (Equal). **Sonya G. Jackson**: Data curation (Equal); Formal analysis (Equal); Methodology (Equal); Visualization (Equal); Writing – original draft (Equal); Writing – review & editing (Equal). **Gabriel Skogberg**: Data curation (Equal); Formal analysis (Equal); Methodology (Equal); Visualization (Equal); Writing – review & editing (Equal). **Anders J. Lundqvist**: Data curation (Equal); Writing – review & editing (Equal). **Ramon Hendrickx**: Data curation (Equal); Formal analysis (Equal); Writing – original draft (Equal); Writing – review & editing (Equal). **Anders Cavallin**: Data curation (Equal); Writing – review & editing (Equal). **Torben Österlund**: Investigation (Equal); Methodology (Equal); Writing – original draft (Equal); Writing – review & editing (Equal). **Neil P. Grimster**: Investigation (Equal); Methodology (Equal); Writing – original draft (Equal); Writing – review & editing (Equal). **Magnus Nilsson**: Conceptualization (Equal); Data curation (Equal); Formal analysis (Equal); Investigation (Equal); Writing – original draft (Equal); Writing – review & editing (Equal). **Annika Åstrand**: Conceptualization (Equal); Data curation (Equal); Formal analysis (Equal); Investigation (Equal); Project administration (Lead); Supervision (Equal); Writing – original draft (Lead); Writing – review & editing (Lead).

## ETHICS STATEMENT

All preclinical procedures were approved by the local Ethical committee in Gothenburg (36/2015) and the approved site number is 31‐5373/11. Human cell experiments were approved by the local ethical committee in Gothenburg (033‐11).

## Supporting information

Supporting Information S1

## Data Availability

Data openly available in a public repository that issues datasets with DOIs.
