# Supplementary material for: Selective Janus kinase 1 inhibition resolves inflammation and restores hair growth offering a viable treatment option for alopecia areata
Source: Skin Health Dis. 2023 Jan 29;3(3):e209. doi: 10.1002/ski2.209 (PMC10233092; doi:10.1002/ski2.209)
Supplement: Supplementary file 1 — Supporting Information S1 [file SKI2-3-e209-s001.docx]

**Supplementary Materials for**

**Selective JAK1 inhibition resolves inflammation and restores hair growth offering a viable treatment option for Alopecia Areata**

J. Mattsson^1^, E. Israelsson^2^, K. Björhall^1^, L. Fahlén Yrlid^1^, K. Thörn^2^, A. Thorén^3^, E. Andersén Toledo^3^, L. Jinton^1^, L. Öberg^2^, C. Wingren^1^, S. Tapani^4^, S.G. Jackson^1^, G. Skogberg^1^, A.J. Lundqvist^5^, R. Hendrickx^5^, A. Cavallin^1^, T. Österlund^6^, N.P. Grimster^7^, M. Nilsson^8^, A. Åstrand^1^*

*Corresponding author: annika.astrand@astrazeneca.com*

**The PDF file includes:**

Figure S1. Taqman bars of 8 selected CTL/IFN genes

Figure S2. Effects on JAK/STAT expression by long-term JAK1 inhibition in mice

Figure S3. Selective JAK1 inhibition demonstrates overall similar immunosuppressive responses as panJAK inhibition with some deviations

Table S1. Clinical observations on hair growth expressed as hair index score (% area x score, 0 to 3)

Table S2. Hypodermal hair follicle number for all mice

Table S3. Mean follicle width for all mice

Table S4. Circulating cytokines in mouse plasma

Table S5. Expression levels of selected CTL/IFN genes in skin expressed as fold change (log_2_) from AA

Detailed Material & Method descriptions where needed incl synthesis of Compound B

**Fig. S1. Taqman bars of 8 selected CTL/IFN genes.** The mRNA expression of three selected cytotoxic lymphocyte (Cd8a, Gzmb, Icos) and five interferon (Cxcl9, Cxcl10, Cxcl11, Stat1, Mx1) signature genes are shown in relation to the expression found in AA animals (Log2 fold change, n=6 per group). Data shown as boxplots around the mean. CD8a and Gzmb was not expressed in healthy and systemic JAK1 inhibition completely suppressed the Gzmb expression.

**
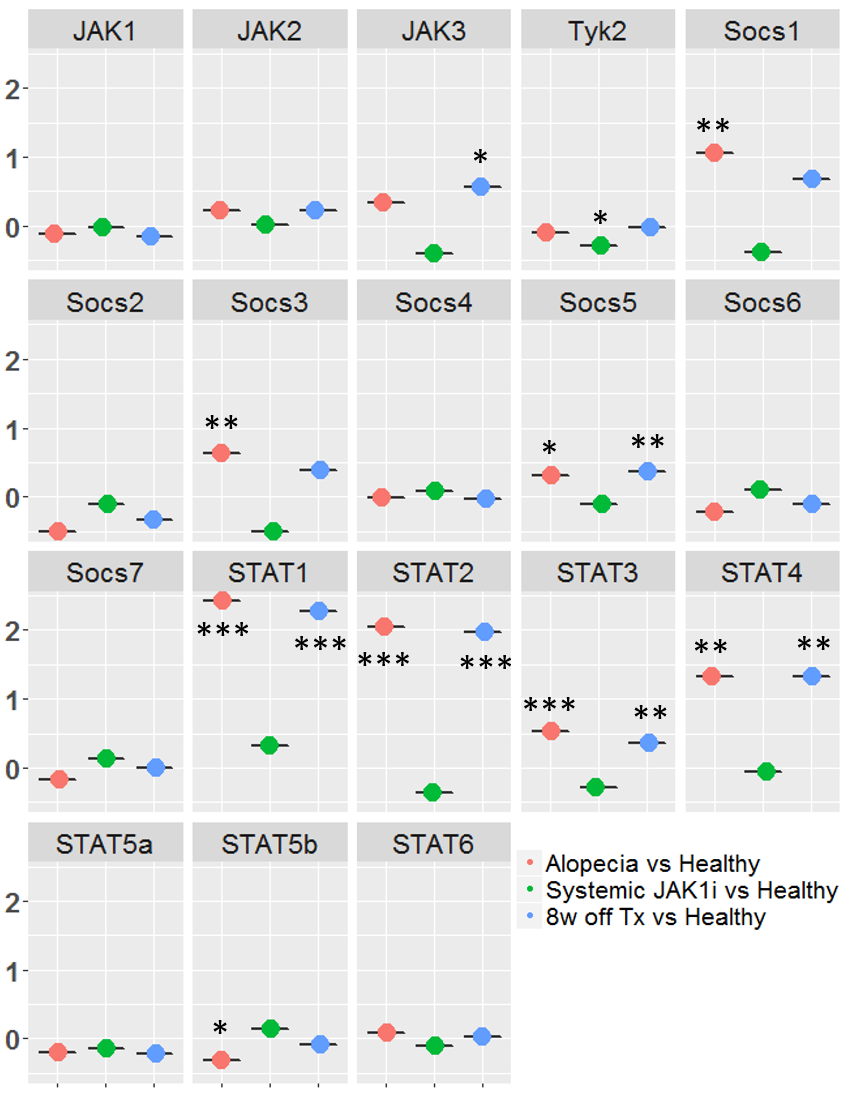
**

**Fig. S2. Effects on JAK/STAT expression by long-term JAK1 inhibition in mice.** Log2 fold change values for a set of selected JAK, STAT, and Socs genes for the Alopecia (pink), Systemic JAK1i (green), and 8w off Tx (blue) groups compared to Healthy. Statistical significance by FDR-corrected p-values are indicated with stars (* FDR< 0.05, ** FDR< 0.01 , *** FDR< 0.001). Log2FC and FDR were obtained from DESeq2.

TNFα

IL-1RA

IL-6

IL-4

IL-10

IP-10

TNFβ

IL-17

IL-1β

MIP-1β

**Figure S3.** **Selective JAK1 inhibition demonstrates overall similar immunosuppressive responses as panJAK inhibition with some deviations.** The levels of secreted mediators from PBMC stimulated with plate bound anti-CD3 in the presence or absence of different JAK inhibitors are shown. DMSO control is set to 100%. Supernatants were collected after 40-48h of stimulation and secreted mediators were analyzed. TNFα (p=0.034 vs Ruxo, p=0.043 vs Tofa), IL-1RA (p=0.0014 vs Ruxo, p=0.030 vs Tofa), IP-10 (p=0.0094 vs Ruxo, 0.0012 vs Tofa), IL-17 (p<0.0001 vs Ruxo and Tofa) and MIP-1β (p=0.031 vs Ruxo, p=0.077 vs Tofa) were significantly differently released by cells treated with the selective JAK1 inhibitor, Cmpd A. Each data point depict Mean+SEM from 4 healthy donors. Statistical analyses was performed by means of linear regression analysis.

**Table S1. Clinical observations on hair growth expressed as hair index score (% area x score, 0 to 3)**

|  | **w0** | **w1** | **w2** | **w3** | **w4** | **w6** | **w7** | **w8** | **w9** | **w10** | **w11** | **w12** |
| --- | --- | --- | --- | --- | --- | --- | --- | --- | --- | --- | --- | --- |
| **Healthy** | 300 |  |  |  | 295 | 295 | 295 | 295 | 295 |  | 295 |  |
|  | 300 |  |  |  | 290 | 290 | 290 | 290 | 290 |  | 290 |  |
|  | 300 |  |  |  | 295 | 295 | 295 | 295 | 295 |  | 295 |  |
|  | 300 |  |  |  | 300 | 300 | 300 | 300 | 300 |  | 300 |  |
|  | 300 |  |  |  | 295 | 295 | 295 | 295 | 295 |  | 295 |  |
|  | 300 |  |  |  | 300 | 300 | 300 | 300 | 300 |  | 300 |  |
| mean±SD | 300±0 |  |  |  | 296±4 | 296±4 | 296±4 | 296±4 | 296±4 |  | 296±4 |  |
| **AA** | 0 |  |  |  | 0 | 0 | 0 | 0 | 0 | 0 | 0 | 0 |
|  | 0 |  |  |  | 0 | 0 | 0 | 0 | 0 | 0 | 0 | 0 |
|  | 0 |  |  |  | 0 | 0 | 0 | 0 | 0 | 0 | 5 | 10 |
|  | 0 |  |  |  | 0 | 0 | 0 | 0 | 0 | 0 | 0 | 0 |
|  | 0 |  |  |  | 0 | 0 | 0 | 0 | 0 | 0 | 0 | 0 |
|  | 0 |  |  |  | 0 | 0 | 0 | 0 | 0 | 0 | 0 | 0 |
| mean±SD | 0±0 |  |  |  | 0±0 | 0±0 | 0±0 | 0±0 | 0±0 | 0±0 | 0.8±2 | 1.7±4 |
| **Systemic** | 0 | 0 | 160 | 280 | 300 |  |  |  |  |  |  |  |
| **JAK1i** | 0 | 0 | 170 | 285 | 300 |  |  |  |  |  |  |  |
|  | 0 | 0 | 180 | 290 | 300 |  |  |  |  |  |  |  |
|  | 0 | 15 | 120 | 250 | 300 |  |  |  |  |  |  |  |
|  | 0 | 10 | 190 | 295 | 300 |  |  |  |  |  |  |  |
|  | 20 | 15 | 160 | 280 | 300 |  |  |  |  |  |  |  |
| mean±SD | 3±8 | 7±7 | 163±24 | 280±16 | 300±0 |  |  |  |  |  |  |  |
| **Systemic** | 125 | 125 | 170 |  | 285 |  |  | 245 |  |  |  | 90 |
| **JAK1i** | 50 | 50 | 200 |  | 295 |  |  | 200 |  |  |  | 60 |
|  | 20 | 20 | 200 |  | 300 |  |  | 210 |  |  |  | 85 |
|  | 50 | 50 | 140 |  | 295 |  |  | 195 |  |  |  | 45 |
|  | 90 | 90 | 180 |  | 295 |  |  | 270 |  |  |  | 205 |
|  | 90 | 90 | 190 |  | 300 |  |  | 75 |  |  |  | 35 |
| mean±SD | 71±38 | 71±38 | 180±23 |  | 295±5 |  |  | 199±67 |  |  |  | 87±62 |
| **Local** | 50 | 50 |  |  | 80 | 200 | 200 | 200 | 240 |  | 240 |  |
| **JAK1i** | 0 | 0 |  |  | 0 | 0 | 80 | 0 | 0 |  | 0 |  |
| **(treated** | 0 | 0 |  |  | 10 | 50 | 0 | 0 | 25 |  | 0 |  |
| **side)** | 0 | 0 |  |  | 0 | 0 | 100 | 0 | 0 |  | 0 |  |
|  | 0 | 0 |  |  | 20 | 0 | 0 | 50 | 70 |  | 100 |  |
|  | 50 | 100 |  |  | 0 | 30 | 100 | 100 | 90 |  | 80 |  |
| mean±SD | 17±26 | 25±42 |  |  | 18±31 | 47±78 | 80±75 | 58±80 | 71±91 |  | 70±94 |  |
| **Local** | 30 | 40 |  |  | 70 | 170 | 170 | 170 | 200 |  | 200 |  |
| **JAK1i** | 0 | 0 |  |  | 0 | 0 | 0 | 0 | 0 |  | 0 |  |
| **(untreated** | 0 | 0 |  |  | 10 | 10 | 0 | 0 | 0 |  | 0 |  |
| **side)** | 0 | 0 |  |  | 0 | 0 | 0 | 0 | 0 |  | 0 |  |
|  | 0 | 0 |  |  | 30 | 0 | 0 | 10 | 25 |  | 30 |  |
|  | 10 | 100 |  |  | 0 | 20 | 0 | 0 | 0 |  | 50 |  |
| mean±SD | 7±12 | 23±41 |  |  | 18±28 | 33±67 | 28±69 | 30±69 | 37±80 |  | 47±78 |  |

Data are shown as mean±SD, n=6 per group.

**Table S2. Hypodermal hair follicle number for all mice**

| **Number of hair follicles per section** | | | | |
| --- | --- | --- | --- | --- |
|  | Healthy | AA | Systemic JAK1i | 8w off Tx |
|  | 4 | 184 | 150 | 51 |
|  | NA | 300 | 542 | 595 |
|  | 0 | 187 | 263 | 35 |
|  | 38 | 583 | 363 | 9 |
|  | 3 | 220 | 20 | 44 |
|  | 0 | 234 | 288 | 48 |
| Mean | 9 | 285 | 271 | 130 |
| St Dev | 16 | 152 | 179 | 228 |
| 95% CI | (-11;29) | (125;444) | (83;459) | (-109;370) |

NA=Not available due to poor quality of the section. Mean±SD. 95% confidence intervals (CI) given in brackets.

**Table S3. Mean follicle width for all mice**

| **Follicle width (μm)** | | | | |
| --- | --- | --- | --- | --- |
|  | Healthy | AA | Systemic JAK1i | 8w off Tx |
|  | 24 | 32 | 28 | 36 |
|  | NA | 29 | 26 | 26 |
|  | NA | 31 | 29 | 38 |
|  | 28 | 28 | 25 | 31 |
|  | 20 | 33 | 26 | 35 |
|  | NA | 33 | 27 | 26 |
| Mean | 24 | 31 | 27 | 32 |
| St Dev | 3.8 | 1.9 | 1.4 | 5.1 |
| 95% CI | (-2.3;31) | (29;33) | (25;28) | (26;37) |

The follicle width is a result of the total measured circular area of all hypodermal follicles per mouse and the total number of follicles in the dermal fat layer. NA=Not available due to poor quality of the section or 0 follicles found.

**Table S4. Circulating cytokines in mouse plasma at 4 weeks**

| **Analyte levels (pg/mL) as measured by Luminex analysis** | | | | | | | | | | | |
| --- | --- | --- | --- | --- | --- | --- | --- | --- | --- | --- | --- |
|  | IFN-γ | IL-1β | IL-6 | TNFα | IL-10 | IL-22 | IL-2 | IL-13 | IL-17A | IL-23 | IL-33 |
| Healthy | <1.0 | <1.9 | <0.8 | 2.4 (±0.4) | 1.5 (±0.3) | 7.9 (±1.2) | 4.5 (±1.0) | 27 (±4.7) | 24 (±6.4) | 712 (±428) | 294 (±328) |
| AA | 50 (±21) | 2.9 (±0.8) | 4.0 (±1.4) | 8.4 (±1.5) | 2.3 (±0.6) | 24 (±9.4) | 5.0 (±0.5) | 42 (±9.7) | 23 (±9.4) | 267 (±130) | 90 (±65) |
| Local  JAK1i | 42 (±14) | 2.4 (±0.5) | 2.8 (±NA) | 7.3 (±0.8) | 2.9 (±1.4) | 35 (±25) | 6.2 (±1.6) | 37 (±2.9) | 15 (±2.4) | 148 (±95) | 76 (±58) |
| Systemic  JAK1i | 49 (±21) | 2.0 (±NA) | 2.9 (±2.3) | 6.3 (±2.0) | 2.4 (±0.7) | 42 (±31) | 4.5 (±1.5) | 33 (±13) | 15 (±4.2) | 206 (±333) | 32 (±17) |

Mean±95% confidence intervals, n=6 per group, NA=not applicable since only one value within detection range.

**Table S5. Expression levels of selected CTL/IFN genes in skin expressed as fold change (log_2_) from AA**

| Gene | AA | Healthy | Systemic JAKi | 8w off Tx | Local JAK1i | Local JAK1i |
| --- | --- | --- | --- | --- | --- | --- |
|  |  |  |  |  | (treated side) | (untreated side) |
| CD8a | 0.00 ± 0.52 | nv | -2.56 ± 0.24  (<0.001, n=6) | -0.32 ± 0.45  (0.97, n=6) | 1.06 ± 0.63  (0.245, n=6) | 1.02 ± 0.71  (0.80, n=6) |
| GZMB | 0.00* ± 0.55 | nv | nv | -0.81 ± 0.73  (0.099, n=3) | 0.93 ± 0.35  (0.45, n=5) | 0.77 ± 0.37  (0.64, n=5) |
| ICOS | 0.00* ± 0.15 | -0.36 ± 0.27  (0.36, n=4) | -1.05 ± 0.12  (0.0083, n=4) | -0.05 ± 0.09  (0.34, n=2) | 1.33 ± 0.18  (<0.001, n=4) | 1.06 ± 0.29  (0.0018, n=5) |
| CXCL9 | 1. ± 0.29 | -4.28 ± 0.49  (<0.001, n=6) | -3.83 ± 0.47  (<0.001, n=6) | -0.33 ± 0.73  (0.96, n=6) | 0.60 ± 0.50  (0.69, n=6) | 0.82 ± 0.65  (0.41, n=6) |
| CXCL10 | 1. ± 0.20 | -3.84 ± 0.43  (<0.001, n=6) | -3.28 ± 0.42  (<0.001, n=5) | -0.32 ± 0.61  (0.97, n=6) | 0.23 ± 0.49  (0.99, n=6) | -0.03 ± 0.74  (0.99, n=6) |
| CXCL11 | 1. ± 0.25 | 0.00 ± 0.32  (0.85, n=4) | -4.32 ± 0.36  (<0.001, n=5) | 0.43 ± 0.73  (0.93, n=6) | 0.06 ± 0.63  (0.99, n=6) | 0.08 ± 0.76  (0.99, n=6) |
| STAT1 | 0.00 ± 0.14 | -2.21 ± 0.28  (<0.001, n=6) | -2.29 ± 0.25  (<0.001, n=6) | -0.30 ± 0.28  (0.67, n=6) | 1.09 ± 0.29  (<0.001, n=6) | 1.07 ± 0.29  (<0.001, n=6) |
| MX1 | 1. ± 0.26 | -2.56 ± 0.19  (<0.001, n=6) | -3.71 ± 0.21  (<0.001, n=6) | -0.37 ± 0.41  (0.67, n=6) | 0.03 ± 0.38  (0.99, n=6) | 0.02 ± 0.41  (0.99, n=6) |
| Krt32 | 0.00 ± 0.20 | -3.25 ± 0.29  (<0.001, n=6) | -3.16 ± 0.13  (<0.001, n=6) | -1.44 ± 0.98  (0.062, n=6) | -0.81 ± 0.70  (0.49, n=6) | -0.64 ± 0.71  (0.70, n=6) |

Log2 fold change (calibr to AA) data. Mean±SEM. P-values in brackets (1-way ANOVA and Dunnett’s post hoc tests to determine statistical significance).

**Detailed M&M section**

***Pharmacokinetics and evaluation of compound exposures:*** Male C57/BL mice were obtained from Si Bei Fu and weighed approximately 20-30 grams (7-9 weeks) at the start of the pharmacokinetic study. Animals had free access to food and water during the course of the experiment. Dose formulations were prepared on the study day and were stirred at room temperature for at least 15 minutes prior dosing and during the administration event. Cmpd A, B and C were dosed at 0.5-1 mg/kg i.v. and 1-300 mg/kg p.o. The dorsal metatarsal vein was used for sampling and typically 0.02 mL blood was withdrawn. Blood was taken at 2, 5, 15, 45 min, 2, 4, 8, 24 hours post the i.v. dose and at 15, 30 min, 1, 2, 4, 8, 24 hours post the p.o. dose. Blood of each sample was transferred into plastic micro centrifuge tubes containing 2 μL of 1000 IU Heparin-Na anticoagulant. These tubes were then inverted several times and placed on wet ice prior to centrifugation at 10,000 rpm for 2 minutes at 4°C in order to collect plasma. Plasma samples were stored at -75±15°C prior analysis. Compound concentrations were analyzed using a LC-MS/MS method (see below).

Satellite PK animals were dosed similarly to the pharmacodynamic (PD) C3H/HeJ animals to produce a PK profile that was used to estimate the average compound exposure based on the occasional PD exposures measured. WinNonlin (PhoenixTM, version 6.1) was used for the pharmacokinetic calculations. Area under the concentration-time curve (AUC_0-tlast_) was estimated using the log-linear trapezoidal method similarly for the area under first moment curve (AUMC_0-tlast_). AUC_0-infinity_ = AUC_0-tlast_ + C_plasma_, t_last_/k, where k is the terminal slope from semi‐log graph of plasma conc. vs time, AUMC_0-infinity_ = AUMC_0-tlast_ + (C_plasma_, t_last_ x t)/k + C _plasma_, t_last_/k^2^. Clearance (CL) was calculated as dose/AUC_0-infinity_, where V_ss_ equals AUMC_0-infinity_ / AUC_0-infinity_ x CL. Effective T_1/2_ equals Ln2 x V_ss_/CL. Finally, %F was calculated as ratio of dose normalized AUC_0-infinity_’s from either administration route multiplied by 100%. %F = 100% x AUC_0-infinity, po_ / AUC_0-infinity, iv_ x IV Dose/PO Dose. The plasma concentrations obtained from the study C3H/HeJ animals were matched to the PK profile to give an estimate of the long-term exposure.

***Plasma and skin exposures of compound A, B, C & Tofacitinib:*** Plasma and skin samples were analyzed by liquid chromatography-tandem mass spectrometry (LC-MS/MS). An Agilent 1290 Infinity II binary LC pump was used with gradient elution using a flow rate at 0.5 ml/min. The mobile phase consisted of (A) 0.2 % formic acid in water and (B) 0.2 % formic acid in acetonitrile. Separation was performed on a 50 x 2.1 mm Poroshell 120 C18-Aq column with 2.7 μm fused core particles (Agilent Technologies Inc., Wilmington, DE, USA) using a linear gradient of 5-95 % B-phase in 1.5 minutes, held at 95 % for 1 minute and returned to initial conditions in one step. The front, containing salt and highly polar compounds, was diverted to waste and after 0.3 minutes the effluent entered the MS. Sample storage and injection was performed by an Agilent 1290 Infinity II Multisampler. Detection was performed by an Agilent 6490 triple quadrupole in multiple reactions monitoring (MRM) positive electrospray ionization mode. The mass transitions were for Compound A, B, C and Verapamil (Internal standard) 489.2 > 127.1, 416.3 > 396.3, 600.2 > 157.2 and 455.3 > 165.0, respectively. The calibration range for the compound was 0.1 nM - 1840 nM. Instrument control, data acquisition and data evaluation were controlled by the software Agilent MassHunter.

Before injection to the LC-MS/MS, 50 μL of plasma samples was protein precipitated in 96 deep well plates using a robot (Bravo, Agilent Technologies Inc., Wilmington, DE, USA) by addition of 180 μL acetonitrile containing an internal standard (Verapamil, 25nM). After vortexing, the samples were centrifuged for 20 minutes at 2900 g and 4 °C. 75 μL of the supernatant was transferred to a new plate and diluted with 75 μL of 0.2 % formic acid in water.
Skin samples were put in inert plastic bags and frozen in liquid Nitrogen. After pulverized by a pneumatic hammer (Covaris) the samples were transferred to 10 mL glass tubes and Ringer´s solution (1 mL) is added. The samples were ultrasonicated for 30 min and further extracted with Acetonitrile (3 mL containing internal standard). After vortexing, the samples were centrifuged for 20 minutes at 2900 g and 4 °C. 75 μL of the supernatant was transferred to a 96 well plate and diluted with 75 μL of 0.2 % formic acid in water.

***RNA extraction:*** Frozen 20 mg skin biopsies were transferred to 2 mL eppendorf tubes placed on dry ice, added 5 stainless steel beads of 3 mm ø and 750 μL cold QIAzol^®^ Lysis Reagent (QIAGEN Sciences, Maryland 20847, USA). The samples were mounted on a Mixer Mill Tissue Lyser (Retsch GmbH, Haan, Germany) and homogenized for 2 x 5 minutes at 30 Hz, then centrifuged for 1 minute at 6000g, added 150 μL CHCl_3_, vigorously shaken for 15 seconds and incubated for 3 minutes. After 15 minutes centrifugation at 6000g the aqueous RNA containing phase was transferred to a 96 deep well plate and mixed with an equal volume of 70 % Ethanol. The RNA-Ethanol precipitate was purified and DNase treated on a 6100 Nucleic Acid Prep Station (Applied Biosystems, Foster City, CA, USA). Total RNA was eluted in 100 μL Elution Buffer (Applied Biosystems, Foster City, CA, USA) and the concentrations were measured on the NanoDrop ND-1000 Spectrophotometer (Thermo Fischer Scientific, Wilmington, DE, USA) at 260 nm.

***cDNA synthesis:*** cDNA synthesis was performed using the High Capacity cDNA Reverse Transcription Kit from Life Technologies (Applied Biosystems, Foster City, CA, USA) according to the manufacturer´s instructions. The cDNA samples were diluted with RNase free water to 100 ng/µL, in a later (experiment) to 200 ng/µL.

***qPCR:*** qPCR was performed on a QuantStudio™ 7 Flex Real-Time PCR System (Applied Biosystems, Foster City, Ca, USA) using custom made Mouse Skin Heat Map TaqMan^®^ Array Micro Fluidic Cards (24 format) as described in the TaqMan^®^ Array Micro Fluidic Cards user guide by Applied Biosystems (ThermoFischer Scientific) with 100 to 200 ng cDNA template. The QuantStudio™ 7 Flex Real-Time PCR System Software was used for the analysis. All thresholds were set to 0.2 and C_T_ values from distorted curves as well as C_T_ values above 32 were excluded from the analysis. Gene expression was calculated using the 2^-ΔΔC^_T_ method. The target genes were normalized to a reference gene panel (GAPDH, β-Actin and HPRT) and the mean Fold Change was calculated using the normalized expression of the untreated group as calibrator. Results are expressed as log_2_ Fold Change to visualize up- as well as down regulations of gene expression.

***qPCR Results:*** Using the 2^-ΔΔC^_T_ method, the data are presented as the Fold Change in gene expression normalized to an endogenous reference gene and relative to a control group ^80^. Here, the geometrical mean C_T_ values from a panel of reference genes (GAPDH, β-Actin and HPRT) were used to normalize the expression of the target genes. The mean Fold Change was calculated using the normalized expression of the (untreated) AA group as the calibrator. For the different treatment groups, evaluation of 2^-ΔΔC^_T_ indicates the Fold Change in gene expression relative to the calibrator group. By definition, the Fold Change of the calibrator is one. In practice, the mean Fold Change will be very close to one. The variance in the mean Fold Change is a consequence of converting the results of an exponential process into a linear comparison of amounts as the standard errors (for the exponentials) are asymmetrically distributed relative to the group average value.

The gene expression results are shown as log_2_ Fold Change. With the calibrator group close to zero, up- or down regulation of the target genes are visualized as positive or negative values, making it easier to assess the size of, in particular, the down regulations.

***Synthesis of Compound B:*** General Synthetic Chemistry. All solvents and chemicals used were reagent grade unless otherwise noted. Anhydrous solvents THF and DCM were purchased from Sigma-Aldrich. Flash column chromatography was carried out using prepacked silica cartridges (from 12 g up to 330 g) and eluted using an ISCO Combiflash Rf system. 1H and 13C NMR spectra were recorded on either a Bruker Avance 300 MHz, a Bruker NEO 500 HMz, or a Bruker nano-AV3HD 400 MHz. 1H chemical shifts are reported in ppm relative to solvent peaks as the internal reference. Splitting patterns are indicated as follows: s, singlet; d, doublet; t, triplet; m, multiplet; br, broad peak. Products were characterized using a Waters ultraperformance liquid chromatography system fitted with both DAD and ELSD detectors and a Waters SQD mass spectrometer. Chromatography was performed using a Waters Acquity HSS T3, 1.8 µm, 2.1 x30 mm column with flow rate = 1 mL/min and a solvent gradient of 2 to 98% B over 1.5 minutes, where A = 0.1% formic acid in water and B = 0.1% formic acid in acetonitrile. Mass spec detection was ESI with positive/negative switching and cone voltage = 10 V. All compounds are >95% pure by ELSD detection.

**5-bromo-3-(2,5-dimethyl-1H-pyrrol-1-yl)-2-fluoropyridine.**

To a solution of hexane-2,5-dione (3.8 mL, 32.7 mmol) in toluene (200 mL) was added 5-bromo-2-fluoropyridin-3-amine (5.0 g, 26.2 mmol), *p*-TSA (0.06 g, 0.3 mmol), and heated to reflux under a Dean-Stark trap. After 2 h, the reaction was allowed to cool to room temperature, then diluted with ethyl acetate and washed with sat. NaHCO_3_ aq., water, and brine. The organic layer was taken, dried over sodium sulfate, filtered and evaporated under reduced pressure. The resultant residue was purified by column chromatography (silica, 15% ethyl acetate/hexanes) to yield the title compound as a yellow oil (6.8 g, 97%); ^1^H NMR (400 MHz, CDCl_3_) δ 8.33 (dd, 1 H), 7.82 (dd, 1 H), 5.94 (s, 2 H), 2.03 (s, 6 H). MS: Calc. for [M+H]^+^ *m/z* 269.0; Obs. 270.0.

**(1*S*,4*S*)-2-(5-(2,5-dimethyl-1H-pyrrol-1-yl)-6-fluoropyridin-3-yl)-5-methyl-2,5-diazabicyclo[2.2.1]heptane.**

To a solution of 5-bromo-3-(2,5-dimethyl-1H-pyrrol-1-yl)-2-fluoropyridine (5.0 g, 18.8 mmol) in 1,4-dioxane (120 mL) was added (1*S*,4*S*)-2-methyl-2,5-diazabicyclo[2.2.1]heptane dihydrobromide (5.1 g, 18.8 mmol), Pd_2_(dba)_3_ (0.9 g, 0.9 mmol), RuPhos (1.7 g, 3.7 mmol), and NaO*t*Bu (8.9 g, 93.0 mmol) and heated to 60 °C. After 45 min, the reaction mixture was filtered through filter agent with 1,4-dioxane. The filtrate was evaporated under reduced pressure, and the resultant residue purified by column chromatography (silica, 0 to 10% MeOH/ DCM) to yield the title compound as a brown gum (1.95 g, 35%); ^1^H NMR (400 MHz, DICHLOROMETHANE-*d2*) δ 7.52 - 7.47 (m, 1 H), 6.85 (dd, *J* = 8.03, 3.01 Hz, 1 H), 5.89 - 5.85 (m, 2 H), 4.14 (s, 1 H), 3.45 (s, 1 H), 3.40 (dd, *J* = 8.78, 2.26 Hz, 1 H), 3.22 (dd, *J* = 8.78, 1.25 Hz, 1 H), 2.82 (dd, *J* = 9.66, 2.13 Hz, 1 H), 2.67 (dd, *J* = 9.66, 1.38 Hz, 1 H), 2.32 (s, 3 H), 2.05 (s, 6 H), 1.86 (dt, *J* = 9.50, 1.10 Hz, 1 H); MS: Calc. for [M+H]^+^ *m/z* 301.1; Obs. 301.1.

**2-fluoro-5-((1*S*,4*S*)-5-methyl-2,5-diazabicyclo[2.2.1]heptan-2-yl)pyridin-3-amine.**

To a solution of (1*S*,4*S*)-2-(5-(2,5-dimethyl-1H-pyrrol-1-yl)-6-fluoropyridin-3-yl)-5-methyl-2,5-diazabicyclo[2.2.1]heptane (1.9 g, 6.5 mmol) in EtOH (90 mL) was added hydroxylamine hydrochloride (9.0 g, 130 mmol) and aqueous hydroxylamine (50%, 8.0 mL, 130 mmol). The mixture was heated to 90 °C. After 16 h, the solution was concentrated under reduced pressure, and the resultant residue triturated with 15% MeOH/DCM, and the solid collected by filtration and purified by column chromatography (silica gel, 0-10% MeOH/DCM) to yield the title compound as a yellow solid (0.91 g, 63%); ^1^H NMR (400 MHz, DMSO-*d6*) δ 6.63 (t, J = 2.89 Hz, 1 H), 6.39 (dd, J = 9.66, 2.64 Hz, 1 H), 5.14 (s, 2 H), 4.11 (s, 1 H), 3.37 (s, 1 H), 3.24 (dd, *J* = 8.78, 2.26 Hz, 1 H), 3.04 (d, *J* = 9.03 Hz, 1 H), 2.73 (dd, *J* = 9.29, 2.01 Hz, 1 H), 2.44 (dd, *J* = 9.54, 1.00 Hz, 1 H), 2.22 (s, 3 H), 1.86 - 1.78 (m, 1 H), 1.72 (m, 1 H); Calc. for [M+H]^+^ *m/z* 223.1; Obs. 223.3.

**N-(2-fluoro-5-((1*S*,4*S*)-5-methyl-2,5-diazabicyclo[2.2.1]heptan-2-yl)pyridin-3-yl)formamide.**

Acetic anhydride (2.9 mL, 31.5 mmol) was added to formic acid (5.1 mL, 134.9 mmol) and the resultant mixture stirred at room temperature. After 30 min, a solution of 2-fluoro-5-((1*S*,4*S*)-5-methyl-2,5-diazabicyclo[2.2.1]heptan-2-yl)pyridin-3-amine (2.0 g, 9.0 mmol) in THF (25 mL) was added, and the resultant reaction mixture stirred at room temperature. After 1 h, the reaction mixture was poured into 2M Na_2_CO_3_ aq. and the resultant mixture extracted with ethyl acetate (x6). The combined organics were dried over sodium sulfate, filtered and evaporated to afford the title compound as a beige solid (2.0 g, 95%); ^1^H NMR (400 MHz, DMSO-*d6*) δ 10.00 - 9.64 (m, 1 H), 8.24 - 8.57 (m, 1 H), 7.77 (br. s, 1 H), 7.21 (t, *J* = 2.76 Hz, 1 H), 4.24 (br. s, 1 H), 3.42 (s, 1 H), 3.31 (dd, *J* = 9.03, 2.26 Hz, 1 H), 3.15 (dd, *J* = 8.91, 0.88 Hz, 1 H), 2.78 (dd, *J* = 9.41, 2.13 Hz, 1 H), 2.53 - 2.50 (m, 1 H), 2.28 (s, 3 H), 1.88 (ddt, *J* = 9.38, 2.29, 1.32, 1.32 Hz, 1 H), 1.75 (dt, *J* = 9.47, 1.04 Hz, 1 H); Calc. for [M+H]^+^ *m/z* 251.1; Obs. 251.1.

**4-Chloro-N-(2-fluoro-5-((1*S*,4*S*)-5-methyl-2,5-diazabicyclo[2.2.1]heptan-2-yl)pyridin-3-yl)-5-methylpyrimidin-2-amine.**

NaH (0.67 g, 16.8 mmol, 60% oil dispersion) was added portionwise to a solution of N-(2-fluoro-5-((1*S*,4*S*)-5-methyl-2,5-diazabicyclo[2.2.1]heptan-2-yl)pyridin-3-yl)formamide (0.7 g, 2.8 mmol) in THF (20 mL) at 0 °C under a nitrogen atmosphere. The mixture was stirred for 10 minutes, then 4-chloro-5-methyl-2-(methylsulfonyl)pyrimidine (0.64 g, 3.1 mmol) was added, and the resultant reaction stirred at 40 °C. After 16 h, the resultant reaction mixture was cooled to 0 °C, and quenched with sat. NaHCO_3_ aq. (Caution - exothermic reaction), then organics were removed under reduced pressure. The resultant mixture was extracted with DCM (x3). The organic layers were combined, dried over sodium sulfate, filtered, and concentrated under reduced pressure. The resultant residue was purified by column chromatography (silica gel, 0-10% MeOH/DCM) to yield the title compound as an off-white solid (0.8 g, 83%); ^1^H NMR (300 MHz, DMSO-*d6*) δ 8.26 (s, 1 H), 8.23 (dd, *J* = 9.03, 3.01 Hz, 1 H), 7.24 (br. s., 1 H), 7.02 (t, *J* = 2.89 Hz, 1 H), 4.17 (s, 1 H), 3.45 (s, 1 H), 3.43 - 3.37 (m, 1 H), 3.28 (dd, *J* = 8.91, 1.13 Hz, 1 H), 2.88 (dd, *J* = 9.54, 2.01 Hz, 1 H), 2.65 (dd, J = 9.54, 1.25 Hz, 1 H), 2.33 (s, 3 H), 2.25 (s, 3 H), 2.01 – 1.94 (m, 1 H), 1.84 - 1.90 (m, 1 H); Calc. for [M+H]^+^ *m/z* 349.1; Obs. 349.1.

-

***tert*-butyl (2*E*)-2-methylbut-2-enoate**.

2-methylprop-1-ene (80 g, 1.4 M) was added to a Parr bomb reactor that had been cooled in a dry ice/acetone bath. After addition was complete, (*E*)-2-methylbut-2-enoic acid (20 g, 0.2 M) and sulfuric acid (1g, 10.2 mmol) were added and the was reaction sealed. The reaction was allowed to warm to room temperature under mechanical stirring (19 PSI). The reaction was stirred at room temperature for 4 days, then the reactor was cooled in a dry ice/acetone bath for 1 hour (0 PSI). The reactor was removed from the dry ice/acetone bath, and the valve was opened and reaction allowed to warm to room temperature. After 2 hours, the reaction vessel was flushed with nitrogen, and then opened. The resultant residue was dissolved in ether, and washed with 10% NaHCO_3_ aq. (x2). The organic layer was taken, diluted with hexane, and washed with 10% NaHCO_3_ aq. and brine. The organic layer was taken dried over sodium sulfate, filtered and evaporated to yield the title compound as a yellow oil (31 g, 100 %). ^1^H NMR (300 MHz, DICHLOROMETHANE-*d2*) δ 6.81 - 6.56 (m, 1H), 1.84 - 1.73 (m, 6H), 1.48 (s, 9H).

***tert*-butyl (2*S*,3*R*)-3-{benzyl[(1*R*)-1-phenylethyl]amino}-2-methylbutanoate**

*n*-butyllithium (38.4 ml, 96.0 mmol) was added dropwise to a solution of (*R*)-N-benzyl-1-phenylethanamine ( 21.6 g, 0.1 mol) in toluene (128 mL) at 0 °C under nitrogen. After addition was complete, the reaction mixture was stirred for 15 minutes at 0 °C, and then cooled to -78 °C. After, *tert*-butyl (2*E*)-2-methylbut-2-enoate (10 g, 64.0 mmol) in toluene (64 mL) was added dropwise, while maintaining internal temperature below -70 °C. The reaction mixture was stirred at -78 °C for 1 hour, and then warmed to -30 °C for 2 hours. The reaction mixture was subsequently cooled to -78 °C, and diluted with THF (640 mL) which had been pre-cooled to -78 °C. The resultant reaction mixture was allowed to and stirred at to -78 °C. After 30 minutes, a solution of 2,6-di-*tert*-butylphenol (39.6 g, 0.2 mol) in THF (64 mL) was added dropwise and the resultant reaction mixture was allowed to warm to room temperature. After 16 h, the reaction was evaporated to dryness and the resultant residue was purified by column chromatography (silica, hexanes 0-10% ethyl acetate/hexanes) to yield the title compound as a yellow oil (17 g, 72 %). ^1^H NMR (300 MHz, DICHLOROMETHANE-*d2*) δ 7.56 - 6.97 (m, 1H), 4.08 - 3.91 (m, 1H), 3.60 - 3.89 (m, 2H), 2.88 (dq, *J* = 9.8, 6.6 Hz, 1H), 2.28 (br dd, *J* = 9.8, 6.8 Hz, 1H), 1.48 - 1.37 (m, 12H), 1.17 - 1.12 (m, 2H), 0.83 (d, J = 7.0 Hz, 3H). Calc. for [M+H]^+^ *m/z* 368.3; Obs. 368.3.

**(2*S*,3*R*)-3-{benzyl[(1*R*)-1-phenylethyl]amino}-2-methylbutan-1-ol.**

LiAlH_4_ (2M in THF, 46.3 mL, 92.5 mmol) was added dropwise to a solution of *tert*-butyl (2*S*,3*R*)-3-{benzyl[(1*R*)-1-phenylethyl]amino}-2-methylbutanoate (17 g, 46.2 mmol) in THF (46.3 ml) at 0 °C under a nitrogen atmosphere. The resultant reaction mixture was allowed to warn to room temp overnight. Sodium hydroxide (2 M, 45 mL) was added dropwise to the reaction mixture with stirring. After 2 h, the reaction mixture was filtered through filter agent, and the filter cake was washed with ether (x4). The organics were combined, washed with water (x2) and brine, then dried over sodium sulfate, filtered and evaporated to yield the title compound as a viscous orange oil (14.1 g, 100%). ^1^H NMR (300 MHz, DICHLOROMETHANE-*d2*) δ 7.49 - 7.15 (m, 10H), 4.13 – 3.96 (m, 1H), 3.94 - 3.83 (m, 1H), 3.80 - 3.68 (m, 2H), 3.46 - 3.32 (m, 1H), 3.19 (br d, *J* = 10.0 Hz, 1H), 2.99 - 2.84 (m, 1H), 1.86 (dt, *J* = 6.8, 3.2 Hz, 1H), 1.44 (d, *J* = 7.0 Hz, 3H), 1.23 - 1.19 (m, 3H), 0.87 (d, *J* = 7.0 Hz, 3H). Calc. for [M+H]^+^ *m/z* 298.4; Obs. 298.3.

**(2*S*,3*R*)-3-amino-2-methylbutan-1-ol HCl.**

(2*S*,3*R*)-3-{benzyl[(1*R*)-1-phenylethyl]amino}-2-methylbutan-1-ol (9.2 g, 30.9 mmol) was dissolved in MeOH (100 mL) and acetic acid (1.3 mL, 23.2 mmol) and palladium hydroxide on carbon (4.3 g, 30.6 mmol) were added. The resultant reaction mixture was sealed under a hydrogen atmosphere (balloon), and the reaction was stirred at room temperature. After 3 hours, the reaction mixture was filtered through filter agent, and the filter cake was washed with methanol. The filtrate was acidified with methanolic HCl (1 M, 50 mL), and evaporated to dryness to yield the title compound as a white solid (4.0 g, 93%). ^1^H NMR (300 MHz, METHANOL-*d4*) δ 3.76 (br dd, *J* = 8.3, 2.8 Hz, 2H), 3.46 - 3.36 (m, 1H), 1.98 (br dd, *J* = 4.1, 2.8 Hz, 1H), 1.34 (d, *J* = 6.8 Hz, 3H), 0.99 (d, *J* = 7.2 Hz, 3H).

**(2*S*,3*R*)-3-{[2-({2-fluoro-5-[(1*S*,4*S*)-5-methyl-2,5-diazabicyclo[2.2.1]heptan-2-yl]pyridin-3-yl}amino)-5-methylpyrimidin-4-yl]amino}-2-methylbutan-1-ol (Compound B).**

(2*S*,3*R*)-3-amino-2-methylbutan-1-ol HCl (0.24 g, 1.7 mmol) was added to a solution of 4-chloro-N-(2-fluoro-5-((1*S*,4*S*)-5-methyl-2,5-diazabicyclo[2.2.1]heptan-2-yl)pyridin-3-yl)-5-methylpyrimidin-2-amine (0.3g, 0.9 mmol) and *N*-ethyl-*N*-isopropylpropan-2-amine (0.45 mL, 2.6 mmol) in *n*-butanol (3 mL). The resulting reaction mixture was sealed and heated in a microwave reactor to 140 °C. After 3 hours, the reaction was cooled to room temperature, filtered and evaporated. The resultant residue was purified by reverse phase preparative HPLC (column Info: CSH C18 4.6mm x 50mm 5μm, mobile phase A: water/TFA: 0.1%, mobile phase B: methanol. Gradient 10 to 40%B over 5 min. Flow rate: 1 mL/min) to yield a gummy solid. The solid was added to sat. K_2_CO_3_ aq. and extracted with ethyl acetate (x3). The organic layers were combined, dried over sodium sulfate, filtered and evaporated to yield compound B (0.13 g, 35%). ^1^H NMR (500MHz, DMSO-*d6*) δ 7.96 - 7.92 (m, 2H), 7.70 - 7.63 (m, 1H), 7.02 (t, *J* = 2.4 Hz, 1H), 6.49 - 6.41 (m, 1H), 5.15 - 4.66 (m, 1H), 4.23 (br s, 2H), 3.47 (br d, *J* = 3.7 Hz, 2H), 3.32 - 3.28 (m, 2H), 3.18 - 3.13 (m, 1H), 2.77 (br d, *J* = 1.5 Hz, 1H), 2.48 (s, 1H), 2.25 (s, 3H), 1.91 (s, 3H), 1.89 - 1.83 (m, 2H), 1.80 - 1.74 (m, 1H), 1.15 (d, *J* = 6.7 Hz, 3H), 0.86 (d, *J* = 6.9 Hz, 3H). Calc. for [M+H]^+^ *m/z* 416.2; Obs. 416.2. ee: >99% determined by HPLC (column Info: Chiralpak IC 4.6 x 250 mm, 5 µm, mobile phase A: hexane/ 0.1% diethylamine, mobile phase B: 1:1 methanol : ethanol/ 0.1% diethylamine, isocratic 20 % B, flow rate: 1 mL/min).

**REFERENCES**

1. Wong P, Pamer EG. Disparate in vitro and in vivo requirements for IL-2 during antigen-independent CD8 T cell expansion. J Immunol. 2004;172(4):2171-6.

2. Livak KJ, Schmittgen TD. Analysis of relative gene expression data using real-time quantitative PCR and the 2(-Delta Delta C(T)) Method. Methods. 2001;25(4):402-8.
